# The repair and autophagy mechanisms of hypoxia‐regulated bFGF‐modified primary embryonic neural stem cells in spinal cord injury

**DOI:** 10.1002/sctm.19-0282

**Published:** 2020-02-06

**Authors:** Sipin Zhu, Min Chen, Liancheng Deng, Jinjing Zhang, Wenfei Ni, Xiangyang Wang, Felix Yao, Xiaokun Li, Huazi Xu, Jiake Xu, Jian Xiao

**Affiliations:** ^1^ Department of Orthopaedics The Second Affiliated Hospital and Yuying Children's Hospital of Wenzhou Medical University Wenzhou China; ^2^ Molecular Pharmacology Research Center School of Pharmaceutical Sciences, Wenzhou Medical University Wenzhou China; ^3^ Molecular Laboratory, School of Pathology and Laboratory Medicine The University of Western Australia Perth Western Australia Australia

**Keywords:** axon regeneration, hypoxia, hypoxia‐responsive elements, neural stem cells, spinal cord injury

## Abstract

There is no effective strategy for the treatment of spinal cord injury (SCI), a devastating condition characterized by severe hypoxia and ischemic insults. In this study, we investigated the histology and pathophysiology of the SCI milieu in a rat model and found that areas of hypoxia were unevenly interspersed in compressed SCI. With this new knowledge, we generated embryonic neural stem cells (NSCs) expressing basic fibroblast growth factor (bFGF) under the regulation of five hypoxia‐responsive elements (5HRE) using a lentiviral vector (LV‐5HRE‐bFGF‐NSCs) to specifically target these hypoxic loci. SCI models treated with bFGF expressed by the LV‐5HRE‐bFGF‐NSCs viral vector demonstrated improved recovery, increased neuronal survival, and inhibited autophagy in spinal cord lesions in the rat model due to the reversal of hypoxic conditions at day 42 after injury. Furthermore, improved functional restoration of SCI with neuron regeneration was achieved in vivo, accompanied by glial scar inhibition and the evidence of axon regeneration across the scar boundary. This is the first study to illustrate the presence of hypoxic clusters throughout the injury site of compressed SCI and the first to show that the transplantation of LV‐5HRE‐bFGF‐NSCs to target this hypoxic microenvironment enhanced the recovery of neurological function after SCI in rats; LV‐5HRE‐bFGF‐NSCs may therefore be a good candidate to evaluate cellular SCI therapy in humans.

## Graphical abstract

Embryonic neural stem cells (NSCs) expressing basic fibroblast growth factor (bFGF) under the regulation of five hypoxia responsive elements (5HRE) using a lentiviral vector (LV‐5HRE‐bFGF‐NSCs) to specifically target these hypoxic loci. SCI models treated with bFGF expressed by the LV‐5HRE‐bFGF‐NSCs viral vector demonstrated improved recovery, increased the survival of neurons, and inhibited autophagy in spinal cord lesions in the rat model due to a reversal of hypoxic conditions post‐injury. Furthermore, improved functional restoration of SCI with neuron regeneration was achieved in vivo accompanied by glial scar inhibition and evidence of axon regeneration across the scar boundary.

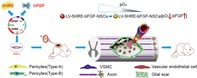

**Significance statement**

The present study shows that application of hypoxia‐regulated basic fibroblast growth factor modified primary embryonic neural stem cells to specifically target the hypoxic loci resulted in a reversal of the hypoxic microenvironment after spinal cord injury (SCI), concomitant with decreased cellular autophagy, reduced CNS glial scar formation, and improved locomotor function in in vivo studies. The results of the present study increase the current understanding of the pathophysiology of SCI and may be used to combat the ischemic microenvironment that can induce cell death and limit cell transplantation approaches to promote spinal cord regeneration.

## 1 INTRODUCTION

Spinal cord injury (SCI) is a devastating event that usually results in significant functional impairment in the patient. SCI is a complex two‐step process in which a cascade of secondary neurodegenerative events is set in motion by a primary injury.^1^ Vascular changes, hypoxia, the loss of ATP‐dependent processes, ionic disturbances, neurotransmitter accumulation, and apoptosis create a toxic milieu, perpetuating the SCI. In an attempt to contain the extent of the damage, the injured central nervous system (CNS) forms a glial scar—a potent physical barrier that prevents axonal regrowth through that region.^2^, ^3^ In an animal model, sustained compression of the spinal cord was shown to lead to a larger histologic lesion and poorer functional outcomes and inhibit the recovery of somatosensory‐evoked potentials.^4^ Effective strategies against this chronic phase of SCI have not been established; clinical trials of nonsurgical treatment options in human SCI have failed to demonstrate marked neurological benefit, in contrast to their success in the laboratory. A better understanding of the events of secondary injury would provide a target to optimize pharmacological and cellular therapies, the timing of surgery, and early rehabilitation.

The pathology behind the mechanism of secondary injury in SCI includes hypoxia and ischemia arising from impaired perfusion at the cellular level and the resulting cellular energy deficiency.^5^ It has been reported that ischemia begins immediately after traumatic SCI and that if not treated, the spinal cord deteriorates in the first 3 hours and continues for at least 24 hours.^6^ However, many studies have also indicated that the spinal cord has a remarkable healing capacity, and early revascularization and oxygenation of the injury are the most important factors to minimize the long‐term, irreversible sequelae of SCI.

Reconstructive and regenerative experimental cellular strategies involving embryonic or adult stem cells or tissue, genetically modified fibroblasts, Schwann cells, olfactory ensheathing cells, and activated macrophages have been reported to exhibit varying degrees of recovery in different models of SCI.^5^, ^7^ A number of growth factors have also been shown to alter different cell types and functions, reducing the deleterious effects of an injury while improving neuronal survival and regeneration.^8^ Angiogenic factors, such as basic fibroblast growth factor (bFGF), which is present in both neuronal and glial cells, have been proposed to address ischemia following injury and were previously reported to have multiple neuropromoting effects on the developing and adult nervous system of mice and other mammals.^9^, ^10^, ^11^ The ischemic environment after SCI leads to limited neuron survival and complicates the transplantation of stem cells designed to promote a permissive environment at the implant site.^12^, ^13^ The implantation of exogenous bFGF or its overexpression in mesenchymal stem cell therapy can spare spinal cord tissue, reduce retrograde degeneration, improve vascularity, and reduce the number of apoptotic cells.^14^, ^15^ However, controlling the release of these factors is a significant challenge because of the potential for increased microvascular permeability associated with an increase in lesion volume and neoplastic development secondary to uncontrolled cell differentiation.^16^ To the best of our knowledge, the delivery of embryonic neural stem cells (NSCs) with the controlled expression of bFGF under the regulation of five hypoxia‐responsive elements (5HRE) using a lentiviral vector (LV‐5HRE‐bFGF‐NSCs) to specifically target hypoxia, and the ischemic microenvironment in SCI has not yet been investigated.

In this study, a rodent model of compressive SCI was established. The expression patterns of hypoxia‐inducible factor‐1α (HIF‐1α), a transacting factor widely expressed in ischemia, were studied at the mRNA and protein levels, and we discovered a pattern of hypoxia with increased cell autophagy throughout the injured spinal cord.^17^ Furthermore, our study shows that the application of LV‐5HRE‐bFGF‐NSCs to specifically target these hypoxic loci reversed the hypoxic microenvironment at day 60 after SCI, concomitant with decreased cellular autophagy, reduced CNS glial scar formation, and improved locomotor function in in vivo studies. The results of this study increase the current understanding of the pathophysiology of SCI and may be employed to combat the ischemic microenvironment, which can induce cell death and limit cell transplantation approaches, to promote spinal cord regeneration.

## 2 MATERIALS AND METHODS

### 2.1 Reagents and antibodies

All reagents we used were commercially available. Fetal bovine serum (FBS) and Dulbecco's modified Eagle's medium (DMEM) were purchased from Invitrogen (Carlsbad, California). Recombinant human bFGF was purchased from Sigma (Sigma‐Aldrich, St. Louis, Missouri). Anti‐GFAP, anti‐bFGF, anti‐p62, anti‐NeuN, and anti‐GAPDH antibodies were purchased from Santa Cruz Biotechnology (Santa Cruz, California). Anti‐GAP43, anti‐LC3, anti‐Beclin‐1, and anti‐Nestin antibodies were purchased from Abcam (CB, United Kingdom). Goat anti‐rabbit and anti‐mouse IgG‐HRP, goat anti‐chicken IgY H&L, donkey anti‐goat IgG H&L were purchased from Santa Cruz Biotechnology. An enhanced chemiluminescence kit and CM‐DiI were purchased from Bio‐Rad (Hercules, California). Thapsigargin (TG) and 3‐methyladenine (3‐MA) were purchased from Sigma‐Aldrich. The autophagy activator rapamycin (RAPA) was purchased from Cell Signaling Technology. All other reagents were purchased from Beyotime Institute of Biotechnology (Shanghai, China) unless otherwise specified.

### 2.2 Isolation and culture of rat embryonic derived NSCs

Wistar rat embryos were obtained at 14‐16 days after pregnancy by routine surgical procedure, followed by separating and immersing the cerebral cortex of each embryo in D‐Hanks solution. The cerebrovascular and meninges were carefully removed under the microscope and centrifuged at 1000 rpm for 3 minutes, the cerebral cortex was then shredded and treated with trypsin (0.125%) and EDTA (0.102%).^18^ Digestion with trypsin/EDTA was terminated by culture medium DMEM/F12 containing 10% FBS, 1% N2 supplements, 2% B27, 20 ng/mL bFGF, 20 ng/mL EGF, 200 IU/L penicillin, and 100 IU/L streptomycin, followed by collecting and centrifuging the cell suspension.^18^ After resuspension with culture medium, cells were transferred into T25 cell culture flasks (0.5 × 10^6^ cells/flask) previously coated with laminin and poly‐ornithine and cultured at 37°C in a moist atmosphere containing 5% CO_2_.

### 2.3 Cell culture and preparation

NSCs were maintained in DMEM supplemented with 10% FBS and 5% horse serum with 2 mM glutamine and penicillin/streptomycin. The cells were cultured in a humidified atmosphere containing 5% CO_2_ and 95% air at 37°C and passaged at 90% confluence. To further evaluate the effect of TG on the survival and proliferation of NSCs, NSCs were treated with TG for 12 hours as previously described. According to our previous study, cells were treated with RAPA (100 nM) or the autophagy inhibitor 3‐MA (5 mM) to further determine the role of autophagy on primary NSCs at 12 hours. All experiments were performed in triplicate.

For grafted cell identification, NSCs were labeled with Cell Tracker CM‐DiI from Invitrogen prior to transplantation by incubating the cells with the dye for 5 minutes at 37°C and another 15 minutes at 4°C. The labeling of the cells with the CM‐DiI dye was verified to be 99%‐100% prior to all transplantations under fluorescent microscope. Cell viability was measured by trypan blue staining at the end of the harvest and before infusion to ensure the viability was greater than 95% for every infusion.

### 2.4 Construction of recombinant lentiviral vector containing bFGF gene

Lentiviral‐based expression vectors LV‐GFP, LV‐bFGF, LV‐5HRE‐GFP, and LV‐5HRE‐bFGF were constructed using the Gateway system (Invitrogen). Full length bFGF, CMV promoter, and GFP sequences were inserted into plasmids. The vectors were obtained with incubation of donors and accepter vectors catalyzed by LR clonase (Gateway LR Clonase Plus Enzyme Mix, Invitrogen). Plasmids were then sequenced to confirm their identity. 293T cells were transfected with plasmids in the presence of lipofectamine 2000 (Invitrogen). After 3 days, the supernatants of culture medium were collected. Viral particles were obtained by ultracentrifugation at 100000*g* for 1 hour and resuspended in DMEM for transduction.

### 2.5 Lentiviral transduction

NSCs were seeded in 24 wells plate at a density of 2 × 10^5^ cells per well. NSCs were exposed to the viral particles in 0.5 mL DMEM at 37°C medium for 4 hours. Cells were transduced with LV‐GFP, LV‐bFGF, LV‐5HRE‐GFP, and LV‐5HRE‐bFGF using polybrene (a final concentration of 8 μg/mL). The medium was then removed, and the cells were washed once with DMEM and then re‐cultured with normal medium with Blasticidin (2 μg/mL) for 14 days. The untransduced cells were eliminated after 14 days by culturing with Blasticidin. Following the stable selection, we found that GFP signal initially observed after transduction was barely detected by confocal analysis, suggestive that IRESs in these constructs were not active in NSCs under the experimental conditions described. Nevertheless, the expression of bFGF was subsequently confirmed by Western blot and confocal analyses as well as enzyme‐linked immunosorbent assay (ELISA).

### 2.6 Enzyme‐linked immunosorbent assay

Cultured supernatants or spinal cord lysates were subjected to ELISA to determine the concentrates of bFGF. For cultured supernatants, cells cultures were treated with cytokines with or without bFGF. Different types of NSCs for ELISA were obtained at 48 hours post‐injury. Tissue samples were homogenized with a dounce tissue grinder in ice‐cold radioimmunoprecipitation assay (RIPA) buffer containing 60 mM NaCl, 1% NP‐40, 0.1% sodium dodecyl sulfate (SDS), 0.5% sodium deoxycholate, and 50 mM Tris‐HCl supplemented with protease inhibitor cocktail (Roche, Mannheim, Germany), followed by centrifuging at 12000 rpm for 10 minutes to obtain supernatants for ELISA. Protein concentrations in the cultured supernatants or tissue homogenates were measured using Micro BCA Protein Assay kits (Pierce, Rockford, Illinois), and equal amounts of samples (typically 10‐20 μg) were loaded into 96‐well plates coated with indicated antibodies. bFGF concentrations were analyzed using bFGF ELISA kits (Institute of Immunology, Tokyo, Japan) according to the manufacturer's instructions.

### 2.7 Animal model of SCI

Eight‐week‐old female Sprague‐Dawley rats weighing 220‐250 g were purchased from the Animal Center of Chinese Academy of Sciences, Shanghai, China. Animals were housed for at least 7 days before the experiment in a room with a 12‐hour light/dark cycle at 23°C‐25°C and received free access to water and food. All protocol of the animal use and care was conducted according to the Guide for the Care and Use of Laboratory Animals from the National Institutes of Health and was approved by the Animal Care and Use Committee of Wenzhou Medical University. All experiments conformed to named local and international guidelines on the ethical use of animals. All the animals were anesthetized by an intraperitoneal injection of 10% chloralic hydras (3.5 mL/kg). Rats were then positioned on a cork platform. An incision in the epidermis along the midline of the back was performed to expose the vertebral column, and a laminectomy was performed at T9 segment of the spine. Moderate crushed injuries were compressed by a vascular clip for 2 minutes (30 g forces, Oscar, China).^15^ Control group animals received the same surgical procedures without impaction. Postoperative nursing contained the artificial emptying of the bladder, twice a day, until the rats restored their bladder function by using cefazolin sodium (50 mg/kg, i.p.).

### 2.8 Transplantation

The animals received transplantation 1 week after SCI. This time point is widely accepted as a suitable therapeutic window as the inflammatory reaction (creating a hostile environment for cell transplant survival) decreases during the first 7 days, and the glial scar that prevents graft host tissue communication is not yet developed.^19^ The animals were fixed in a stereotaxic instrument with a rat‐specific vertebra‐holder (Cunningham spinal adaptor, Stoelting Co., Wood Dale, Illinois), receiving exposure at T9 of spine. A total of 1 × 10^6^ NSCs cells /5 μL (either unlabeled or labeled with CM‐DiI) were injected through 10 μL microinjector (26G, an inner diameter of 0.24 mm, an outer diameter of 0.6 mm, 30° bevel, 1‐cm long needle) into the epicenter at a depth of 1 mm below the dorsal surface at a rate of 1 μL/min using a Nano‐Injector (Stoelting Co.). Cells were obtained as described above, and the culture medium suspension was prepared before transplantation. The number of cells was determined based on our pilot study, in which 2 × 10^5^ NSCs cells /1 μL were also injected into the proximal, central and distal parts of the injured spinal cord. The microinjector was kept in place after injection for another 5 minutes to prevent cell suspension leakage. The control group received 5 μL of phosphate‐buffered saline (PBS).

### 2.9 Behavioral recovery evaluation

In order to define recovery features after SCI, behavioral analyses were conducted by trained investigators who were blind to the experimental conditions. Basso‐Beattie‐Bresnahan (BBB) locomotion scale, inclined plane test, and footprint were performed as described elsewhere to evaluate open‐field locomotion.^20^ BBB locomotion scale is a 22‐point scale (scores 0‐21) that logically and systematically follows recovery of injured hind limb function from a score of 0, showing complete paralysis of lower limbs, to a score of 21, representative of a normal locomotion rodent. The scale was developed based upon natural progression of locomotion recovery in rats with thoracic SCI.

The inclined plane test was performed by a testing apparatus.^21^ The maximum angle at which a rat could retain its position for 5 seconds without falling was recorded for indicated position and averaged to obtain a single score for each animal. Footprint analysis was conducted through dipping the animal's hind limbs into blue dye as described elsewhere,^21^, ^22^ followed by recording and analyzing walking path through a narrow box (1 m long and 7 cm wide).

The average of six values was used for statistical evaluation. The data are expressed as mean ± SEM. All data were compared between the sham operated group and the transplanted group by a two sample *t*‐test for independent samples, if the two samples had equal variances. If they had unequal variances, the Mann‐Whitney test was used for evaluation. A *P*‐value <.05 was considered statistically significant. All behavioral tests were performed by two independent blind observers.

### 2.10 Hematoxylin‐eosin staining and Nissl staining

The rats were anesthetized with 10% chloral hydrate (3.5 mL/kg, i.p.) at 1 month and 2 months after injury, and perfused with 0.9% NaCl, followed by perfusing with 4% paraformaldehyde in 0.01 M PBS (pH = 7.4). The spinal cords from the C1‐L5 segments were excised and fixed in 4% paraformaldehyde overnight at 4°C, and then embedded in paraffin. Transverse paraffin sections (5 μm thick) were mounted on poly‐l‐lysine‐coated slides for hematoxylin‐eosin (H&E) staining or Nissl staining. Images were captured by a light microscope.

### 2.11 Immunofluorescence staining

Animals were perfused through the heart with cold saline followed by 4% paraformaldehyde in 0.1 M phosphate buffer (pH 7.2). The spinal cords were collected and postfixed in 4% paraformaldehyde at 4°C overnight, then transferred to 30% sucrose for 3 days. Horizontal sections of 3.5‐cm long containing the lesion/transplant site were sectioned on a cryostat set at 5‐μm thickness.^23^ Coronal sections from rostral and caudal of spinal cord to the T9 lesion/transplant site were sectioned. The nonspecific binding sites of tissue sections were blocked by incubating with 10% normal serum donkey in PBS containing 0.1% Triton X‐100 at room temperature for 1 hour, followed by incubating with indicated primary antibodies overnight at 4°C. The nuclei were stained with Hoechst 33258 (0.25 μg/mL) dye. For NeuN (1:500), CD31 (1:50), GAP43 (1:1000), bFGF (1:300), LC3 (1:500), and nestin (1:1000) detection in neurons, the different primary antibodies were used. Subsequently, sections were washed four times PBS at room temperature for 10 minutes each time, followed by incubating with Alexa‐Fluor 488/594 donkey anti‐rabbit/mouse, Alexa Fluor 594/647 donkey anti‐mouse/rabbit, or Alexa‐Fluor 488/594 donkey anti‐goat secondary antibody (1:500; Invitrogen Corporation, Carlsbad, California) for 1 hour at room temperature. Sections were then washed four times with PBS containing 0.1% Triton X‐100 at room temperature for 10 minutes each time, followed by three times with PBS for 5 minutes each time and briefly with water. All images were captured by Nikon ECLIPSE Ti microscope (Nikon, Tokyo, Japan).

### 2.12 Western blot analysis

For the in vivo protein analysis, spinal cord segment (0.5 cm length) at the contusion epicenter was isolated and rapidly stored at −80°C for Western blot assays. For protein extraction, the tissue was homogenized in modified RIPA buffer (50 mM Tris‐HCl, 1% NP‐40, 20 mM DTT, 150 mM NaCl, pH = 7.4) containing protease inhibitor cocktail (10 μL/mL, GE Healthcare Biosciences, Pennsylvania). The complex was then centrifuged at 12000 rpm, and the supernatant was obtained for protein assay. In vitro, NSCs were lysed in RIPA buffer (25 mM Tris‐HCl, 150 mM NaCl, 1% Nonidet P‐40, 1% sodium deoxycholate, and 0.1% SDS) with protease and phosphatase inhibitors. The extracts above were quantified with bicinchoninic acid reagents (Thermo, Rockford, Illinois). A total of 50 μg proteins were loaded on a 11.5% SDS‐PAGE gel and then transferred onto a Polyvinylidene fluoride membrane (Bio‐Rad, Hercules, California). The membrane was then blocked with 5% milk (Bio‐Rad) in Tris Buffered saline (TBS) with 0.05% Tween 20 for 1 hour at room temperature. Next, the membranes were incubated with the following antibodies overnight at 4°C: anti‐bFGF (1:300), anti‐GAP43 (1:500), anti‐Beclin‐1 (1:500), anti‐LC3 (1:1000), anti‐P62 (1:1000), and GAPDH (1:1000). The membranes were then washed three times with TBS for 10 minutes each time and incubated with horseradish peroxidase‐conjugated secondary antibodies for 1 hour at room temperature. Plot signals were visualized by ChemiDoc XRS+ Imaging System (Bio‐Rad), and density of each band was quantified with Multi Gauge Software of Science Lab 2006 (FUJIFILM Corporation, Tokyo, Japan). The relative densities of the bands were analyzed by Quantity One (version 4.5.2; Bio‐Rad).

### 2.13 3‐MA and RAPA preparation and treatment

3‐MA (Sigma‐Aldrich) and RAPA (100 nM; Cell Signaling Technology) were first dissolved in Dimethyl sulfoxide (DMSO) (25 mg/mL) and then diluted in PBS for a final dose before i.p. injection. According to our previous study, rats received i injection of 3‐MA (2.5 mg/kg/day), 3‐MA and RAPA (0.5 mg/kg/day), or an equivalent volume of vehicle immediately after SCI. Animals were treated uniformly until the final analysis of the data. All experimental animals received daily rehabilitation, including passive mobilization of the hind legs twice daily. Subsequently, the rats were sacrificed 14 days after injury.

### 2.14 Cell viability assay

NSCs, LV‐GFP‐NSCs, LV‐bFGF‐NSCs, LV‐5HRE‐GFP‐NSCs, and LV‐5HRE‐bFGF‐NSCs were seeded on 96‐well plates (5 × 10^3^ cells/well) and treated with different concentrations of TG (0, 1 μM, 2.5 μM, 5 μM, 10 μM, 15 μM) for 12 and 24 hours to simulate SCI. Cell viability was measured by MTT assay (3‐[4,5‐dimethylthiazol‐2‐yl]‐2,5‐diphenyltetrazolium bromide; 5 mg/mL). During the last 4 hours, MTT was added to the culture medium. Cells were then washed with PBS (pH 7.4), followed by adding DMSO to solubilize the formazan crystals. The absorbance was measured at 570 nm, determining 5 μM TG was optimal concentration for the subsequent experiments. All experiments were performed in triplicate to ensure accuracy.

### 2.15 Apoptosis assay

The apoptotic rates of the NSCs, LV‐GFP‐NSCs, LV‐bFGF‐NSCs, LV‐5HRE‐GFP‐NSCs, and LV‐5HRE‐bFGF‐NSCs treated with TG (5 μM), RAPA (100 nM), RAPA and 3‐MA (5 mM), or 3‐MA were measured using a PI/Annexin V‐FITC kit (Invitrogen) and then analyzed by the FACScan flow cytometer (Becton Dickinson, Franklin Lakes, New Jersey) as described in the manufacturer's manual. All experiments were performed in triplicate.

### 2.16 Statistical analysis

All data were expressed as the mean ± SEM. Statistical significance was determined with Students' *t* test when there were two experimental groups. For more than two groups, statistical evaluation of the data was performed using one‐way analysis of variance test, followed by Dunnett's post hoc test. Values of *P* < .05 were considered significant.

## 3 RESULTS

### 3.1 Generation of embryonic NSCs with the targeted expression of bFGF via the control of a hypoxia‐inducible system

Previous observations prompted us to employ embryonic NSCs expressing bFGF via the control of hypoxia‐inducible conditions (LV‐5HRE‐bFGF‐NSCs) in an attempt to turn the hypoxic loci due to hypoxia‐inducible bFGF expression.

We chose bFGF because bFGF is a known neuroprotectant against a number of brain and SCI conditions.

**Figure 1:**
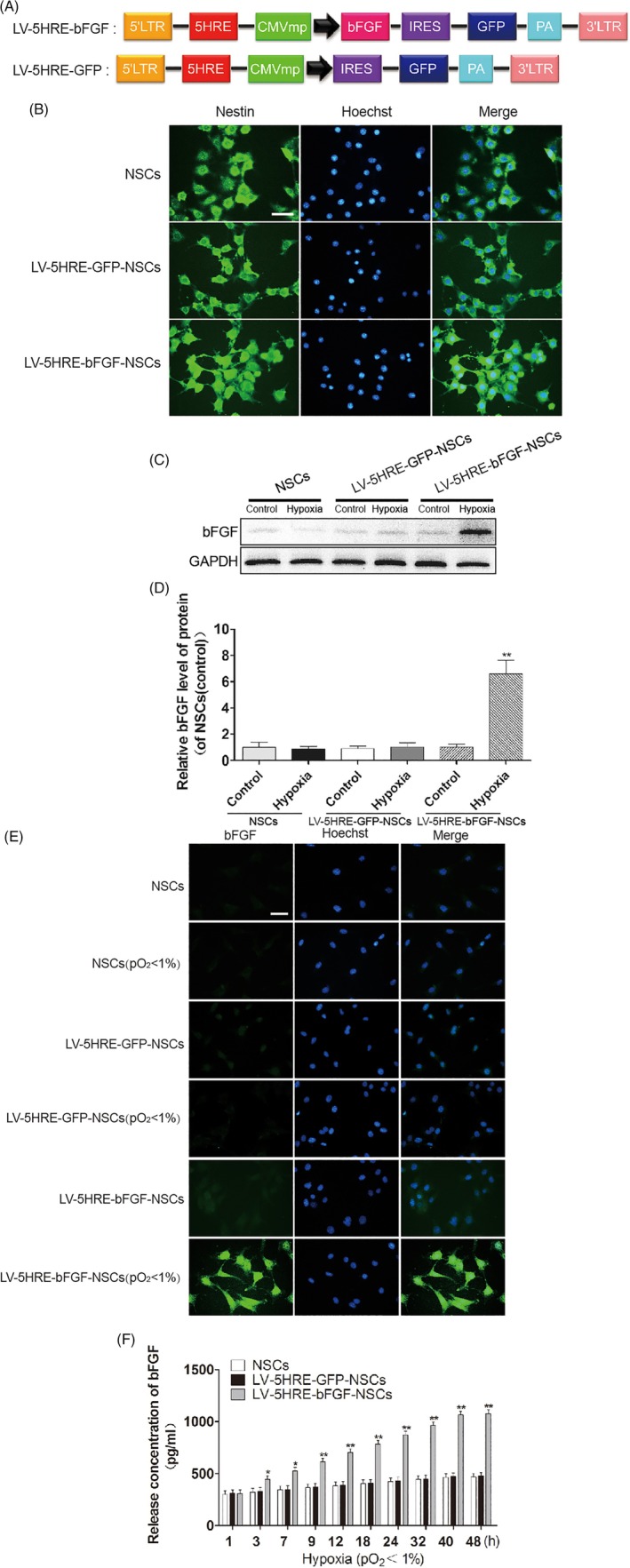
Preparation and characterization of the LV‐5HRE‐GFP‐NSCs, LV‐5HRE‐bFGF‐NSCs. A, Schematic drawing of the lenti/5HRE‐bFGF and lenti/5HRE‐hrGFP vector constructs. B, Nestin staining for the identification of primary neural stem cells successfully generated. Scale bar = 100 μm. C, Under normal and hypoxic conditions, primary NSCs, LV‐5HRE‐GFP‐NSCs, and LV‐5HRE‐bFGF‐NSCs were successfully generated showing bFGF expression by Western blotting under normal or hypoxia (pO2 < 1%) condition for 24 hours. D, The optical density analysis of bFGF protein. ** represents *P* < .01 vs various other groups, data are the mean values ± SEM. E, Under an inducible expression system, LV‐5HRE‐bFGF‐NSCs, but not NSCs, LV‐5HRE‐GFP‐NSCs and LV‐5HRE‐bFGF‐NSCs, showed induced bFGF (green) expression with hypoxia (pO_2_ < 1%) in vitro. Scale bar = 50 μm. F, Levels of bFGF analyzed by ELISA at 0‐48 hours with cultures of NSCs, LV‐5HRE‐GFP‐NSCs, and LV‐5HRE‐bFGF‐NSCs group with hypoxia (pO_2_ < 1%) in vitro. * represents *P* < .05 and ** represents *P* < .01 vs the NSCs and LV‐5HRE‐GFP‐NSCs group, data are the mean values ± SEM. All experiments were repeated three times. 5HRE, five hypoxia‐responsive elements; bFGF, basic fibroblast growth factor; NSC, neural stem cell

We generated NSCs expressing bFGF using a lentivirus system. Under noninducible conditions (Figure S1A), LV‐bFGF‐NSCs exhibited higher expression levels of bFGF than LV‐GFP‐NSCs and NSCs (Figure S1B‐D), and bFGF expression levels increased steadily over time (Figure S1E). Under an inducible expression system (Figure 1A), LV‐5HRE‐bFGF‐NSCs showed induced bFGF expression under hypoxic (pO_2_ < 1%) conditions in vitro, whereas LV‐5HRE‐bFGF‐NSCs and NSCs did not (Figure 1B‐F). Thus, we reasoned that hypoxia‐inducible bFGF overexpression can provide NSCs with favorable conditions for the functional recovery of SCI after their transplantation in vivo.

### 3.2 LV‐bFGF‐NSCs resist TG‐induced apoptosis

**Figure 2:**
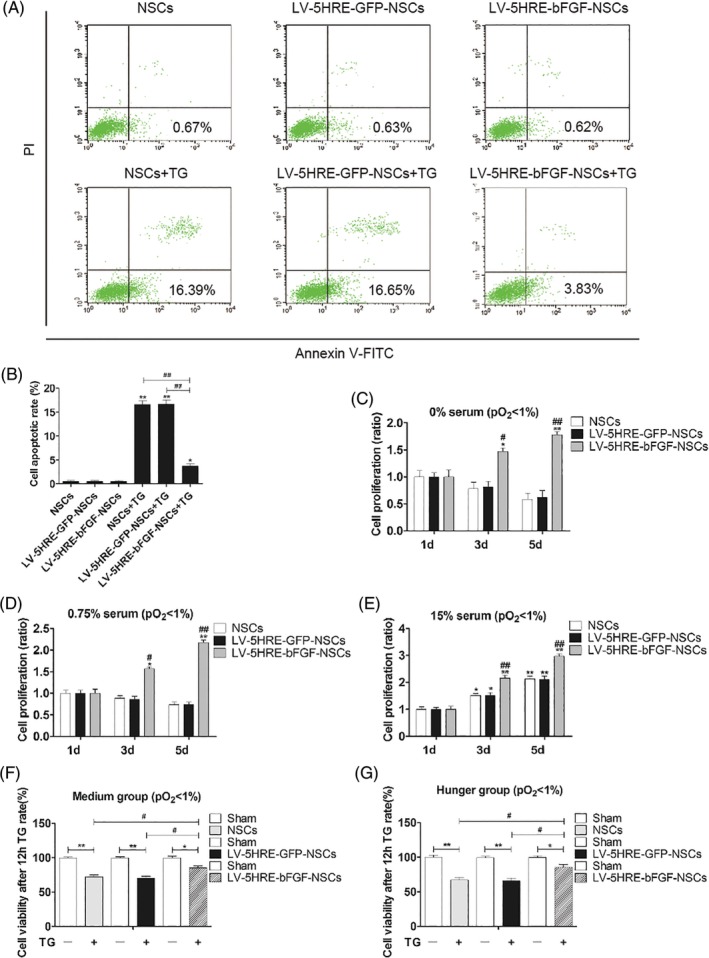
LV‐5HRE‐bFGF‐NSCs increase neural stem cell survival and proliferation in vitro. A, Representative FACS analysis showing PI/Annexin V‐FITC staining apoptotic cells induced by TG for 12 hours with hypoxia (pO_2_ < 1%) in vitro. Values represent the apoptosis rate statistics. B, Percentage of apoptotic cells induced by TG. C‐E, At different concentrations of serum in culture, LV‐5HRE‐bFGF‐NSCs show increased proliferation of neural stem cells by MTT assay in hypoxic conditions (pO_2_ < 1%). Especially at low concentrations or free serum conditions, the proliferation rate is more significant in LV‐5HRE‐bFGF‐NSCs. * represents *P* < .05 and ** represents *P* < .01 vs the 1d group, # represents *P* < .05 and ## represents *P* < .01 vs NSCs and LV‐5HRE‐GFP‐NSCs group. Data are the mean values ± SEM. F‐G, MTT assay results of different experimental groups treated with TG for 12 hours in vitro. * represents *P* < .05 or ** represents *P* < .01, # represents *P* < .05. Data are the mean values ± SEM. All experiments were repeated three times. 5HRE, five hypoxia‐responsive elements; bFGF, basic fibroblast growth factor; NSC, neural stem cell

TG can induce the apoptosis of NSCs, which is similar to the endoplasmic reticulum stress‐induced apoptosis that occurs in the spinal cord following traumatic SCI. in vitro experiments showed that LV‐bFGF‐NSCs were superior in protecting against TG‐induced apoptosis (Figure S2A‐B) and increased cell proliferation in both serum‐containing and serum‐starved settings under conditions that were not hypoxia‐inducible (Figure S2C‐G). In addition, LV‐5HRE‐bFGF‐NSCs protect against TG‐induced apoptosis (Figure 2A‐B) and induced cell proliferation (Figure 2C‐G) in serum‐containing and serum‐starvation settings under hypoxia‐inducible conditions. We reasoned from our in vitro studies that the hypoxia‐induced overexpression of bFGF can provide NSCs with a superior microenvironment for the functional recovery of SCI after their transplantation in vivo.

### 3.3 Maximizing recovery after SCI by LV‐5HRE‐bFGF‐NSCs

**Figure 3:**
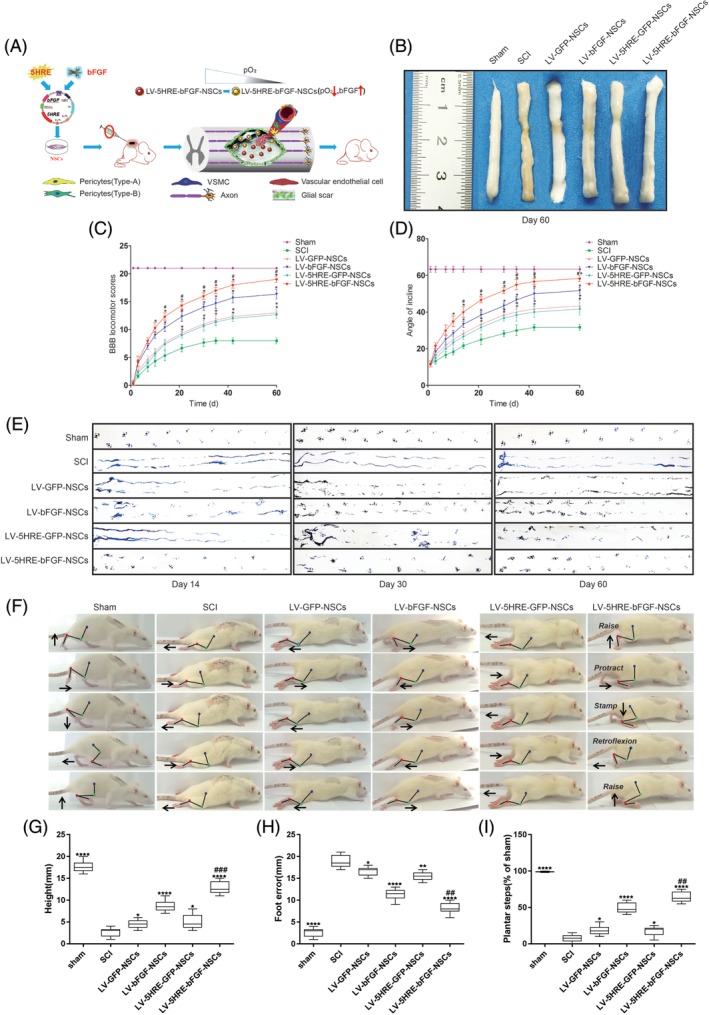
Hypoxic regulated expression of bFGF in primary embryonic neural stem cells maximizes the promotion of spinal cord injury (SCI) repair. A, Schematic flowchart of this study. B, Anatomical features of sham group, SCI group, LV‐GFP‐NSCs group, LV‐bFGF‐NSCs group, LV‐5HRE‐GFP‐NSCs group, and LV‐5HRE‐bFGF‐NSCs group transplantation in SCI, at 60 days after SCI. C, The BBB scores of the sham group, SCI group, LV‐GFP‐NSCs group, LV‐bFGF‐NSCs group, LV‐5HRE‐GFP‐NSCs group, and LV‐5HRE‐bFGF‐NSCs group. The score of sham group was 21 points, which means normal locomotion. D, The inclined plane test scores of the different groups. * represents *P* < .05 vs the SCI group, # represents *P* < .05 vs the LV‐bFGF‐NSCs group. Data are the mean values ± SEM, n = 6. E, Footprint analyses of the different groups in 14, 30, and 60 days. F, Video image sequence of a rat walking 60 days after receiving an SCI. Impaired hind limb function was significantly manifested as poor weight support (quantified as torso height above ground), slow steps (number of hind limb plantar steps per step‐cycle of front leg) and poor foot placement (caudal to behind hip). Iliac crest, knee, and ankle joints are indicated by dots and lines. Arrow shows foot movement. G‐I, Plots of average locomotor parameters for each rat with spinal cord injury (3‐5 weeks after injury), including body height, foot‐placement errors, and plantar steps. 5HRE, five hypoxia‐responsive elements; BBB, Basso‐Beattie‐Bresnahan; bFGF, basic fibroblast growth factor; NSC, neural stem cell

The treatment of SCI with LV‐5HRE‐bFGF‐NSCs dramatically improved the anatomical appearance of injured spinal cords (Figure 3A,B). The mean BBB scores and angle of incline scores used to assess locomotor skills over time were higher in the LV‐5HRE‐bFGF‐NSCs group than in the other five groups. Rats treated with LV‐5HRE‐bFGF‐NSCs also showed a significant improvement in footprint analysis at days 14, 30, and 60 (Figure 3C‐E). Interestingly, locomotor scores in the LV‐5HRE‐bFGF‐NSCs group were distinguishable from those in the other groups as early as 3 days after implantation.

**Figure 4:**
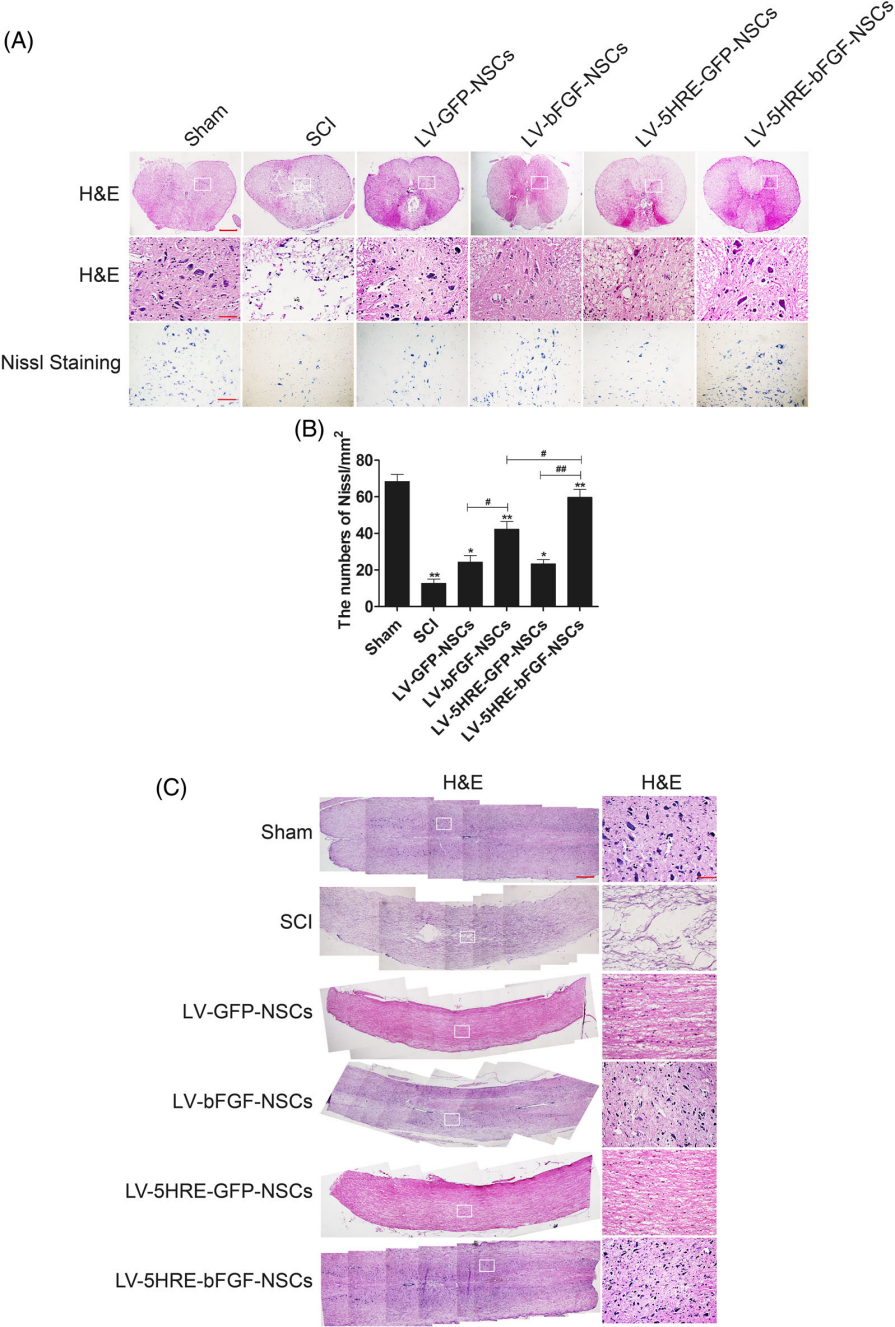
LV‐5HRE‐bFGF‐NSCs have maximizing capacity to fill tissue cavities, promote tissue and nerve repair in SCI. A, H&E staining (cross section) results for the sham group, SCI group, LV‐GFP‐NSCs group, LV‐bFGF‐NSCs group, LV‐5HRE‐GFP‐NSCs group, and LV‐5HRE‐bFGF‐NSCs group, scale bar = 500 μm. A boxed region illustrates a representative region with high power images, scale bar = 100 μm. Nissl staining of the different groups, scale bar = 100 μm. B, Analysis of the Nissl staining results. * represents *P* < .05 and ** represents *P* < .01 vs SCI group. # represents *P* < .05 and ## represents *P* < .01. Data are the mean values ± SEM, n = 3. C, H&E staining (longitudinal section) results for the sham group, SCI group, LV‐GFP‐NSCs group, LV‐bFGF‐NSCs group, LV‐5HRE‐GFP‐NSCs group, and LV‐5HRE‐bFGF‐NSCs group, scale bar = 500 μm. A boxed region illustrates a representative region with high power images, scale bar = 100 μm. 5HRE, five hypoxia‐responsive elements; bFGF, basic fibroblast growth factor; NSC, neural stem cell; SCI, spinal cord injury

Histological analysis of both longitudinal and coronal SCI sections showed that defective areas due to SCI were markedly reduced in the LV‐5HRE‐bFGF‐NSCs group (Figure 4A‐C), with an increased number of Nissl‐positive cells and NeuN‐positive cells and fewer differentiated cell types compared with other groups containing bFGF and GFP. Progressive destruction of the dorsal white matter and central gray matter tissue was found in the SCI group but not the sham operation group. Compared with the SCI group, the LV‐5HRE‐bFGF‐NSCs group showed significant protective effects, as evidenced by less necrosis and karyopyknosis and fewer infiltrated polymorphonuclear leukocytes and macrophages. Notably, transplanted NSCs had differentiated to become neuronal cells over time. We observed that the LV‐5HRE‐bFGF‐NSCs group displayed the highest percentage of differentiated cells among all treatment groups at day 60 (Figure S3A‐C). In addition, the LV‐5HRE‐bFGF‐NSCs group showed the highest percentage of NeuN‐positive cells (Figure S4A‐C). Collectively, these results indicate that transplanted NSCs in the LV‐5HRE‐bFGF‐NSCs group preferentially differentiated into neuronal cells over time within the more favorable microenvironment, maximizing the functional restoration of SCI.

### 3.4 LV‐5HRE‐bFGF‐NSCs showed expanded axon regeneration over the scar boundary, accompanied by astrocyte scar inhibition and increased NSC migration, proliferation, and differentiation

**Figure 5:**
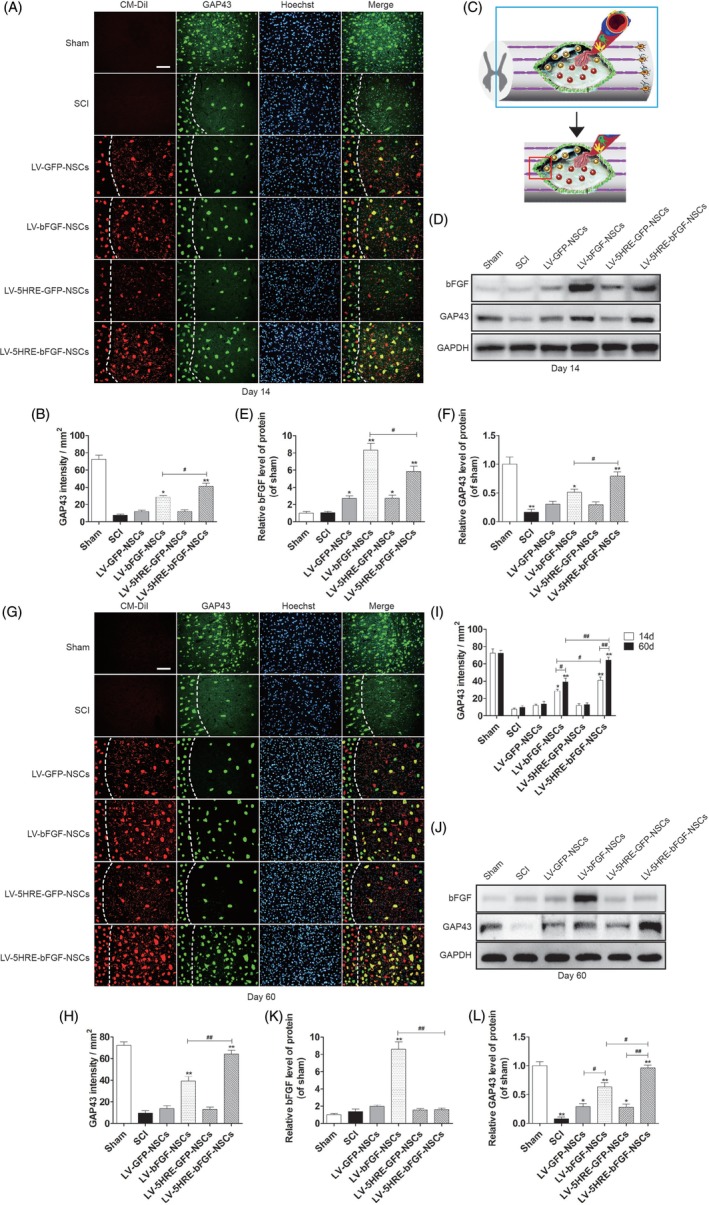
Hypoxia regulated LV‐5HRE‐bFGF‐NSCs maximize the expression of GAP43 protein. A, The representative images of the GAP43 immunostaining the longitudinal spinal cord section from an animal in sham group, SCI group, LV‐GFP‐NSCs group, LV‐bFGF‐NSCs group, LV‐5HRE‐GFP‐NSCs group, and LV‐5HRE‐bFGF‐NSCs group illustrating lesion morphology and transplant at 14 days after SCI. Transplanted NSCs expressing red fluorescent signals (CM‐DiI). Green fluorescence represents GAP43. The nuclear is labeled by Hoechst (blue). The dotted line indicates a presumptive lesion border. Scale bar = 100 μm. B, Quantification of GAP43 staining intensity per unit area. C, Immunofluorescence staining of the longitudinal section of spinal cord injury border. D, The protein expressions of bFGF and GAP43 in the sham group, SCI group, LV‐GFP‐NSCs group, LV‐bFGF‐NSCs group, LV‐5HRE‐GFP‐NSCs group, and LV‐5HRE‐bFGF‐NSCs group were analyzed by Western blotting in 14 days. GAPDH was used as the loading control and for band density normalization. E and F, The optical density analysis of bFGF and GAP43 protein. G, The representative images of the GAP43 immunostaining the longitudinal spinal cord section from an animal in sham group, SCI group, LV‐GFP‐NSCs group, LV‐bFGF‐NSCs group, LV‐5HRE‐GFP‐NSCs group, and LV‐5HRE‐bFGF‐NSCs group illustrating lesion morphology and transplant at 60 days after SCI. Transplanted NSCs express red fluorescent signals (CM‐DiI). Green fluorescence represents GAP43. The nuclear is labeled by Hoechst (blue). The dotted line indicates a presumptive lesion border. Scale bar = 100 μm. H, Quantification of GAP43 staining intensity per unit area. I, Comparison of 14 and 60 days GAP43 immunofluorescence. J, The protein expressions of bFGF and GAP43 in the sham group, SCI group, LV‐GFP‐NSCs group, LV‐bFGF‐NSCs group, LV‐5HRE‐GFP‐NSCs group, and LV‐5HRE‐bFGF‐NSCs group were analyzed by Western blotting in 60 days. GAPDH was used as the loading control and for band density normalization. K and L, The optical density analysis of bFGF, GAP43 protein. * represents *P* < .05 and ** represents *P* < .01 vs the SCI group, # represents *P* < .05 and ## represents *P* < .01. Data are the mean values ± SEM, n = 3. 5HRE, five hypoxia‐responsive elements; bFGF, basic fibroblast growth factor; NSC, neural stem cell; SCI, spinal cord injury

We then examined the expression of growth associated protein 43 (GAP43), a crucial component of the axon that plays a key role in axonal regeneration and plasticity.^24^, ^25^ GAP43 was expressed at the highest level in the LV‐5HRE‐bFGF‐NSCs group on days 14 and 60 (Figure 5A‐L) after transplantation. By confocal analysis, we observed that GAP43 expression signals (green) were detected in almost >90% of transplanted NSCs (red plus green) and some residual NSCs (green only). Notably, GAP43‐positive transplanted NSCs (yellow) migrated beyond the scar (white) from the injury site to normal regions (Figure 5A and G), and this migration was most obvious in the LV‐5HRE‐bFGF‐NSCs group.

**Figure 6:**
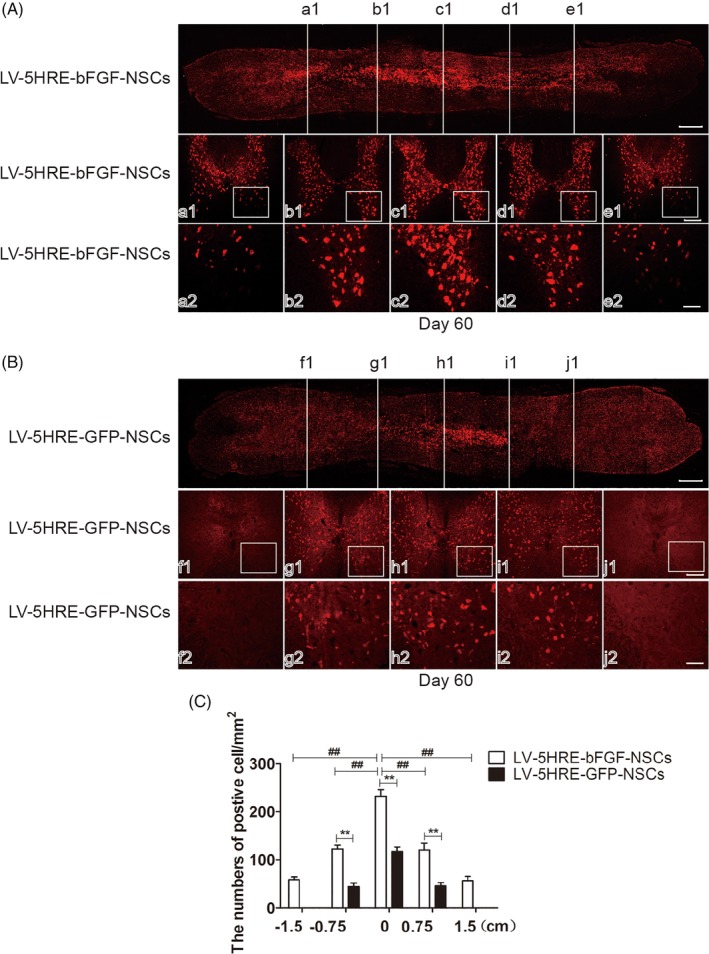
LV‐5HRE‐bFGF‐NSCs promote neural stem cells from the spinal cord injury (SCI) center to both sides. A, LV‐5HRE‐bFGF‐NSCs transplantation into the SCI center. Horizontal section immunolabeled with CM‐DiI signals indicates that implanted cells survive well and distribute through the lesion cavity, scale bar = 500 μm. Rostral is to left, and caudal is to right. White cross lines represent the five cross‐sections of SCI. a1‐e1, Five white lines corresponding to the cross‐sectional view of the spinal cord, scale bar = 200 μm. a2‐e2, A boxed region illustrates a representative region with high power images, scale bar = 100 μm. B, LV‐5HRE‐GFP‐NSCs transplantation into the SCI center. Horizontal section immunolabeled with CM‐DiI signals indicates that implanted cells survive well and distribute through the lesion cavity, scale bar = 500 μm. Rostral is to left, and caudal is to right. White cross lines represent the five cross sections of SCI. a1‐e1, Five white lines corresponding to the cross sectional view of the spinal cord, scale bar = 200 μm. a2‐e2, A boxed region illustrates a representative region with high power images, scale bar = 100 μm. C, Quantification of CM‐DiI labeled positive after transplantation of neural stem cells per mm^2^. ** represents *P* < .01, ## represents *P* < .01. Data are the mean values ± SEM, n = 3. 5HRE, five hypoxia‐responsive elements; bFGF, basic fibroblast growth factor; NSC, neural stem cell; SCI, spinal cord injury

Finally, cross sections of the SCI were examined histologically, and CM‐DiI labeling allowed the migration of NSCs from the initial transplantation site to be easily observed (Figure 6A‐C and Figure S5A‐C). More NSCs in the LV‐5HRE‐bFGF‐NSCs group survived, and NSCs in the 5HRE‐bFGF‐NSCs group migrated better than NSCs in the other groups. These results indicate that the administration of LV‐5HRE‐bFGF‐NSCs could promote spinal cord regeneration, impair glial scar formation, promote axon regeneration across the scar boundary, and enhance neurological function recovery in rats after SCI.

### 3.5 Genetic modification of bFGF to promote the migration of NSCs

Anatomical analysis revealed that grafted cells assessed 60 days after grafting had completely and consistently filled the lesion cavity (Figure 3A‐B). Grafted cells had migrated into the host beyond the immediate region of the graft/lesion site. In addition, CM‐DiI labeling directly showed the NSCs transplant center in the damaged spinal cord, and migration was observed on both sides of the transplant center, with the greater number of CM‐DiI‐labeled cells observed at the heart of the injury, and a gradual decrease in CM‐DiI labeling observed along both sides of the injury. Compared with the GFP‐NSCs group, the BFGF‐NSCs group migrated farther (Figure 4A‐C). These results show that expression of the bFGF protein can promote NSC transplantation and the survival, proliferation, and differentiation of cells in the SCI area to replace the damaged nerve tissue.

### 3.6 Reduction in autophagy and cellular apoptosis induced by LV‐5HRE‐bFGF‐NSCs

**Figure 7:**
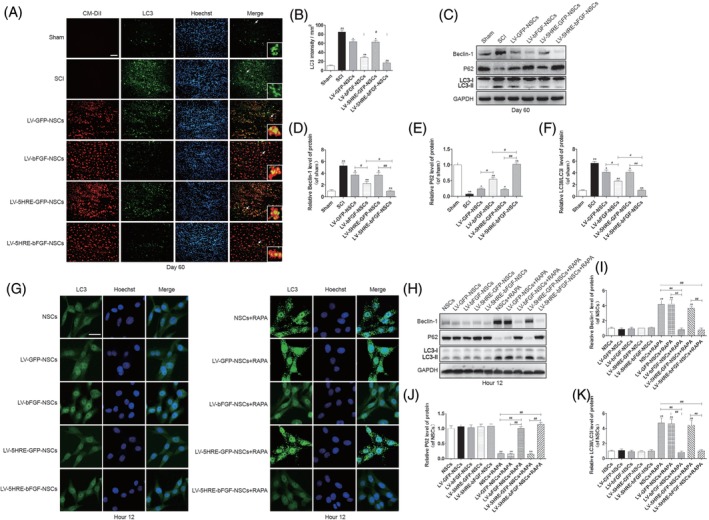
Hypoxia regulated LV‐5HRE‐bFGF‐NSCs reduce autophagy. A, The representative images of LC3 immunostaining in the longitudinal spinal cord section from an animal in sham group, SCI group, LV‐GFP‐NSCs group, LV‐bFGF‐NSCs group, LV‐5HRE‐GFP‐NSCs group, and LV‐5HRE‐bFGF‐NSCs group, illustrating lesion morphology and transplant at 60 days after SCI. Transplanted NSCs express red fluorescent signals (CM‐DiI). Green fluorescence represents LC3. The nuclear is labeled by Hoechst (blue). Scale bar = 200 μm. B, Quantification of LC3 staining intensity per unit area. C, The protein expressions of Beclin‐1, P62 and LC3 in sham group, SCI group, GFP‐NSCs group, LV‐bFGF‐NSCs group, 5HRE‐GFP‐NSCs group, and LV‐5HRE‐bFGF‐NSCs group in 60 days. GAPDH was used as the loading control and for band density normalization. D‐F, The optical density analysis of Beclin‐1, P62, and LC3 protein. * represents *P* < .05 and ** represents *P* < .01 vs the SCI group, # represents *P* < .05 and ## represents *P* < .01, data are the mean values ± SEM, n = 3. G, Rapamycin treatment of NSCs under normal or hypoxia (pO2 < 1%) condition for 12 hours, the immunofluorescence staining results of LC3 in vitro. Green fluorescence represents LC3. The nuclear is labeled by Hoechst (blue). Scale bar = 20 μm. H, The protein expressions of Beclin‐1, P62, and LC3 were analyzed by Western blotting in 12 hours after rapamycin treatment. GAPDH was used as the loading control and for band density normalization. I‐K, The optical density analysis of Beclin‐1, P62, and LC3 protein. ** represents *P* < .01 vs the NSCs group, LV‐GFP‐NSCs group, and LV‐5HRE‐GFP‐NSCs group, ## represents *P* < .01, data are the mean values ± SEM, all experiments were repeated three times. 5HRE, five hypoxia‐responsive elements; bFGF, basic fibroblast growth factor; NSC, neural stem cell; SCI, spinal cord injury

Both in vitro and in vivo studies revealed that the addition of LV‐5HRE‐bFGF‐NSCs to the injured spinal cords of rats reduced apoptosis and RAPA‐induced cellular autophagy under hypoxic conditions, as shown by decreased Beclin‐1 and LC3I/LC3II expression and enhanced p62 expression (Figure 7A‐K and Figure S6A‐D). A similar pattern of Beclin‐1, LC3I/LC3II, and p62 expression was found in all groups that received operation with the addition of 3‐MA, which has been widely used as an autophagy inhibitor based on its inhibitory effect on autophagy activity (Figures S6E‐H and S7A‐J). Interestingly, the treatment of injured rat spinal cords with 3‐MA alone improved the functional restoration of SCI in vivo (Figure S7K and S7L). We observed that RAPA‐induced autophagy‐related apoptosis was protected by 3‐MA administration or LV‐bFGF‐NSCs or LV‐5HRE‐bFGF‐NSCs transplantation in vitro, indicating that both 3‐MA and bFGF have protective effects on autophagy (Figure S8A‐G). Cell viability decreased as the TG concentration increased, whereas combination treatment with bFGF partially increased cell viability compared with that of the NSC + TG group. All these data both in vitro and in vivo demonstrated that the protective role of bFGF in cellular apoptosis is related to the activation of autophagy, contributing to the functional restoration of SCI.

## 4 DISCUSSION

The normal architecture of the human spinal cord is disrupted by injury. SCI leads to a progressive decrease in spinal cord blood flow, leading to ischemic hypoxia and rapid tissue destruction initiated by a cascade of biochemical reactions. First, following the primary traumatic insult, a variety of inflammatory and cytotoxic mediators is released at the injured site, resulting in secondary damage to the spinal cord. Within 24‐72 hours, residing astrocytes become hypertrophic in response to SCI, and the reactive astrocytes produce chondroitin sulfate proteoglycans that form a dense impenetrable scar at the injury site within 7‐14 days.^26^ The glial scar can protect intact neural networks from further damage; however, it also serves as an impediment for regenerating axons as they attempt to reach their distal targets.^27^, ^28^ In addition, myelin‐associated inhibitors and factors expressed by oligodendrocytes have been suggested to play adverse roles in CNS axon regeneration, adding to the inhibitory environment following SCI for neural regeneration.^29^, ^30^ Progressive expansion of the injury across more than one segment results in the formation of microcavities and cysts (syringomyelia), causing significant morbidity and mortality to the affected person.

It has previously been documented that the pathophysiology of SCI is heterogeneous in cause and outcome.^23^, ^31^ To the best of our knowledge, the injured spinal canal displays severe ischemia and hypoxia. In this study, a compressed SCI injury model was used; compressed SCI, a progressive injury that enlarges over tie, normally represents approximately 40% of SCI cases in humans. Hypoxic loci were associated with the increased expression of HIF‐1α (a transcriptional regulator of oxygen homeostasis). This was accompanied by an increase in autophagy activity within these oxygen‐deprived areas, decreasing the number of cells expressing the neuronal biomarker NeuN.^32^

Cell‐based therapies have promising potential for promoting recovery following SCI.^33^ In numerous preclinical studies, stem cells, growth factors, vectors, or a combination of all three have been shown to improve neurological function. However, the survival, fate, and migratory response of transplanted systems and their resultant capacity for neurorepair and functional recovery are likely influenced by factors in the injured microenvironment. Our novel observations enabled us to utilize these hypoxic loci to control bFGF expression and specifically target hypoxic areas. This study highlights a previously undescribed approach for maximizing the recovery from SCI in rats by the successful construction of a lentiviral vector that enables the regulation of bFGF expression by hypoxia, a pathophysiological stimulus, and shows that hypoxia‐induced bFGF expression is neuroprotective. This approach allows bFGF to target the right place at the right time and has the therapeutic potential to treat spinal cord injuries via a timely improvement in the microenvironment at the SCI. To this end, we propose a new working model. Under noninducible conditions, bFGF can be expressed in any cell, albeit at the highest level, driving the proliferation of both “healthy and sick” cells. In contrast, under hypoxia‐inducible conditions, bFGF is expressed in only hypoxic cells that are selectively provided to patients or areas of the most need, creating the most favorable microenvironment for the functional restoration of SCI. When the hypoxic environment in the isolated foci is improved, bFGF expression then returns to its normal levels.

With our increased understanding of the hypoxic SCI milieu, we undertook further investigations to increase the target specificity and improve the safety and efficacy of the expression system used to combat SCI. Animals received transplanted cells 7 days after SCI—after the initial inflammatory infiltration had decreased and bFGF upregulation had peaked and prior to formation of the glial scar that prevents graft‐host tissue communication.^19^ Remarkably, hypoxic conditions were most prominent on day 14 after SCI, as shown by the highest level of HIF‐1α expression. This was accompanied by increased glial scar formation. The combination of NSC transplantation with bFGF expression in the right place at the right time will likely reduce glial scar formation.

The following several lines of evidence support the observation that LV‐5HRE‐bFGF‐NSCs assisted in the regeneration of neurons after SCI in this study:

1. LV‐5HRE‐bFGF‐NSCs reversed the hypoxia‐induced milieu of SCI at day 42 after transplantation and returned the microenvironment to conditions of normoxia at day 60.
2. LV‐5HRE‐bFGF‐NSCs were superior in protecting against cellular apoptosis and autophagy in vitro and in vivo, induced cell proliferation under serum deprivation, and optimized the differentiation of NSCs.
3. Histological analysis showed that CM‐Dil‐labeled NSCs in the LV‐5HRE‐bFGF‐NSCs groups migrated across the scar boundary.
4. A marked improved in the anatomical appearance of injured spinal cords after treatment with LV‐5HRE‐bFGF‐NSCs was associated with superior locomotor functional recovery. Importantly, we failed to observe the same level of neuron regeneration and locomotor function restoration unless each of the above mechanisms that individually contribute to SCI degeneration was targeted.

## 5 CONCLUSION

We report the novel finding of unevenly interspersed hypoxic conditions with increased cell autophagy throughout the injury site of SCI in rats. Established injuries require a better appreciation of altered neuropathophysiology, which will be a determining factor in the restoration of function consequent to successful neuron regeneration by cellular strategies. In this study, we constructed LV‐5HRE‐bFGF‐NSCs, which were successful in simultaneously targeting multiple neural mechanisms responsible for regeneration failure. Improved functional outcomes resulting from neuron regeneration will most likely require advances in each of these mechanisms.

## CONFLICT OF INTEREST

The authors indicated no potential conflicts of interest.

## AUTHOR CONTRIBUTIONS

S.Z.: coordinated and carried out most of the experiments and data analysis, drafting of the manuscript, final approval of manuscript; M.C., L.D., J.Z.: provided technical assistance, including viral vector constructions and cell transfection, final approval of manuscript; W.N., X.W., F.Y.: data analysis and interpretation, revision of the manuscript, final approval of manuscript; X.L., H.X., J. Xu, J. Xiao: supervised the project, experimental design, financial support, final approval of manuscript.

## Supporting information


**Figure S1** Preparation and characterization of the LV‐GFP‐NSCs and LV‐bFGF‐NSCs. **A)** Schematic drawing of the lenti‐bFGF and lenti‐hrGFP vector constructs. **B)** Primary NSCs, LV‐GFP‐NSCs and LV‐bFGF‐NSCs were successfully generated showing bFGF expression by western blotting. **C)** The optical density analysis of bFGF protein. ** represents *P* < 0.01 vs the NSCs group and LV‐GFP‐NSCs group, data are the mean values ± SEM. All experiments were repeated three times. **D)** Nestin staining for the identification of primary neural stem cells successfully generated. Scale bar = 100 μm. **E)** Levels of bFGF analyzed by ELISA at 0‐48 hours with cultures of NSCs, LV‐GFP‐NSCs, LV‐bFGF‐NSCs group in vitro. * represents *P* < 0.05 and ** represents *P* < 0.01 vs the NSCs and LV‐GFP‐NSCs group, data are the mean values ± SEM. All experiments were repeated three times.
**Figure S2.** LV‐bFGF‐NSCs increase neural stem cell survival and proliferation in vitro. **A)** Representative FACS analysis showing PI/Annexin V‐FITC staining apoptotic cells induced by TG for 12 hours in vitro. Values represent the apoptosis rate statistics. **B)** Percentage of apoptotic cells induced by TG. **C‐E)** At different concentrations of serum in culture, LV‐bFGF‐NSCs show increased proliferation of neural stem cells by MTT assay. Especially at low concentrations or free serum conditions, the proliferation rate is more significant in LV‐bFGF‐NSCs. * represents *P* < 0.05 and ** represents *P* < 0.01 vs the 1d group, # represents P < 0.05 and ## represents P < 0.01 vs NSCs and LV‐GFP‐NSCs group. Data are the mean values ± SEM. **F‐G)** MTT assay results of different experimental groups treated with TG for 12 hours in vitro. * represents *P* < 0.05 or ** represents *P* < 0.01, # represents *P* < 0.05. Data are the mean values ± SEM. All experiments were repeated three times.
**Figure S3.** In spinal cord injury, LV‐5HRE‐bFGF‐NSCs maximize the promotion of primary embryonic neural stem cell differentiation and cell survival. **A)** Immunofluorescence staining results of nestin, 60 days after NSCs transplantation in SCI. Transplanted NSCs express red fluorescent signals (CM‐DiI). Green fluorescence represents nestin. The nuclear is labeled by Hoechst (blue). The arrows indicate the typical morphology of nestin. A boxed region illustrates a representative region with high power images. Scale bar = 100 μm. **B)** Immunofluorescence staining of the cross‐section of spinal cord injury center. **C)** Quantification of the percentage of differentiated cells by nestin staining verse CM‐DiI signals. * represents *P* < 0.05 and ** represents *P* < 0.01, # represents *P* < 0.05. Data are the mean values ± SEM, n = 3.
**Figure S4.** In spinal cord injury, LV‐5HRE‐bFGF‐NSCs maximize the promotion of primary embryonic neural stem cell survival and neuronal differentiation. **A)** Immunofluorescence staining results of NeuN, 60 days after NSCs transplantation in SCI. Transplanted NSCs express red fluorescent signals (CM‐DiI). Green fluorescence represents NeuN. The nuclear is labeled by Hoechst (blue). The arrows indicate the typical morphology of NeuN. A boxed region illustrates a representative region with high power images. Scale bar = 100 μm. **B)** Immunofluorescence staining of the cross‐section of spinal cord injury center. **C)** Quantification of the percentage of NeuN positive cells verse CM‐DiI signals. * represents *P* < 0.05 and ** represents *P* < 0.01, # represents *P* < 0.05. Data are the mean values ± SEM, n = 3.
**Figure S5.** LV‐bFGF‐NSCs promote neural stem cells from the spinal cord injury center to both sides. **A)** LV‐bFGF‐NSCs transplantation into the spinal cord injury center. Horizontal section immunolabeled with CM‐DiI signals indicates that implanted cells survive well and distribute through the lesion cavity, scale bar = 500 μm. Rostral is to left, and caudal is to right. White cross lines represent the five cross‐sections of spinal cord injury. (a1‐e1) Five white lines corresponding to the cross sectional view of the spinal cord, scale bar = 200 μm. (a2‐e2) A boxed region illustrates a representative region with high power images, scale bar = 100 μm. **B)** LV‐GFP‐NSCs transplantation into the spinal cord injury center. Horizontal section immunolabeled with CM‐DiI signals indicates that implanted cells survive well and distribute through the lesion cavity, scale bar = 500 μm. Rostral is to left, and caudal is to right. White cross lines represent the five cross‐sections of spinal cord injury. (f1‐j1) Five white lines corresponding to the cross sectional view of the spinal cord, scale bar = 200 μm. (f2‐j2) A boxed region illustrates a representative region with high power images, scale bar = 100 μm. **C)** Quantification of CM‐DiI labeled positive after transplantation of neural stem cells per mm^2^. ** represents *P* < 0.01, ## represents *P* < 0.01. Data are the mean values ± SEM, n = 3.
**Figure S6.** Hypoxia regulated LV‐5HRE‐bFGF‐NSCs minimize the inhibition of autophagy. **A)**The protein expressions of Beclin‐1, P62 and LC3 treated with sham group, SCI group, LV‐GFP‐NSCs group, LV‐bFGF‐NSCs group, LV‐5HRE‐GFP‐NSCs group and LV‐5HRE‐bFGF‐NSCs group in 14 days after SCI. GAPDH was used as the loading control and for band density normalization. **B‐D)** The optical density analysis of Beclin‐1, P62 and LC3 protein. * represents *P* < 0.05 and ** represents *P* < 0.01 vs SCI group, # represents P < 0.05 and ## represents P < 0.01, data are the mean values ± SEM, n = 3. **E)** The protein expression of Beclin‐1, P62, LC3II/LC3I in the different neural stem cell groups treated rapamycin (RAPA), and with RAPA and 3‐methyladenine (3‐MA) under normal or hypoxia (pO_2_ < 1%) condition for 12 hours. **F‐H)** The optical density analysis of Beclin‐1, P62 and LC3 protein. * represents *P* < 0.05 and ** represents *P* < 0.01 vs the NSCs+RAPA group, LV‐GFP‐NSCs+RAPA group and LV‐5HRE‐GFP‐NSCs+RAPA group, ## represents P < 0.01, data are the mean values ± SEM. The data represent the results of three separate experiments.
**Figure S7.** Inhibition of autophagy promotes functional recovery of spinal cord injury. **A)** Immunofluorescence staining results of LC3 after 3‐MA or rapamycin (RAPA) plus 3‐MA treatment for 14 days in SCI. Green fluorescence represents LC3. The nuclear is labeled by Hoechst (blue). Scale bar = 200 μm. **B)** The protein expressions of Beclin‐1, P62 and LC3 in 14 days after SCI. GAPDH was used as the loading control and for band density normalization. **C‐E)** The optical density analysis of Beclin‐1, P62 and LC3 protein. ** represents *P* < 0.01, data are the mean values ± SEM, n = 3. **F)** Immunofluorescence staining results of NSCs treated with RAPA or RAPA plus 3‐MA in vitro. Red fluorescence represents LC3. The nuclear is labeled by Hoechst (blue). Scale bar = 20 μm. **G)** The protein expressions of Beclin‐1, P62 and LC3 in 12 hours after SCI. GAPDH was used as the loading control and for band density normalization. **H‐J)** The optical density analysis of Beclin‐1, P62 and LC3 protein. ** represents *P* < 0.01, data are the mean values ± SEM, All experiments were repeated three times. **K)** The BBB scores of sham group, SCI group, SCI + 3‐MA group and SCI + 3‐MA + RAPA group. The score of sham group was 21 points, which means normal locomotion. * represents *P* < 0.05 vs the SCI group. Data are the mean values ± SEM, n = 6. **L)** The inclined plane test scores of the different groups. * represents P < 0.05 vs the SCI group. Data are the mean values ± SEM, n = 6.
**Figure S8.** RAPA‐induced apoptosis was significantly reduced by 3‐MA and by the expression of bFGF in primary neural stem cells. **A)** FACS result by PI/annexin V‐FITC staining for cell apoptosis analysis treated with RAPA, and RAPA compound with 3‐MA, and 3‐MA for 12 hours under normal or hypoxia (pO_2_ < 1%) condition. Values represent the apoptosis rate. **B‐G)** Statistical result of apoptosis rates. ** represents *P* < 0.01. Data are the mean values ± SEM. All experiments were repeated three times.

## Data Availability

The data used to support the findings of this study are available from the corresponding author upon request.
