# Supplementary material for: The repair and autophagy mechanisms of hypoxia‐regulated bFGF‐modified primary embryonic neural stem cells in spinal cord injury
Source: Stem Cells Transl Med. 2020 Feb 6;9(5):603–19. doi: 10.1002/sctm.19-0282 (PMC7180297; doi:10.1002/sctm.19-0282)

**The repair and** **autophagy mechanisms of hypoxia-regulated bFGF modified primary embryonic neural stem cells in spinal cord injury**

Sipin Zhu ^a, b, c^, Min Chen ^a, b^, Liancheng Deng ^b^, Jinjing Zhang ^b^, Wenfei Ni ^a^, Xiangyang Wang ^a^, Felix Yao ^c^, Xiaokun Li ^b^, Huazi Xu ^a^, Jiake Xu ^a, c^, Jian Xiao ^a, b*^

a Department of Orthopaedics, The Second Affiliated Hospital and Yuying Children’s Hospital of Wenzhou Medical University, Wenzhou, Zhejiang, 325027 China

b Molecular Pharmacology Research Center, School of Pharmacy, Wenzhou Medical University, Wenzhou, Zhejiang, 325035 China

c Molecular Laboratory, School of Pathology and Laboratory Medicine, The University of Western Australia, Perth 6009, M504, Australia

**Correspondence**

Jian Xiao, School of Pharmacy, Wenzhou Medical University, Wenzhou 325035, Zhejiang, PR China.

Tel: +86-577-85773087

Fax: +86-577-85773087

E-mail: [xfxj2000@126.com](mailto:xfxj2000@126.com)

**Supplemental data**

**Supplementary Figure 1.** Preparation and characterization of the LV-GFP-NSCs and LV-bFGF-NSCs. **A)** Schematic drawing of the lenti-bFGF and lenti-hrGFP vector constructs. **B)** Primary NSCs, LV-GFP-NSCs and LV-bFGF-NSCs were successfully generated showing bFGF expression by western blotting. **C)** The optical density analysis of bFGF protein. ** represents P < 0.01 versus the NSCs group and LV-GFP-NSCs group, data are the mean values ± SEM. All experiments were repeated three times. **D)** Nestin staining for the identification of primary neural stem cells successfully generated. Scale bar = 100 μm. **E)** Levels of bFGF analyzed by ELISA at 0-48 hours with cultures of NSCs, LV-GFP-NSCs, LV-bFGF-NSCs group *in vitro*. * represents *P* < 0.05 and ** represents *P* < 0.01 versus the NSCs and LV-GFP-NSCs group, data are the mean values ± SEM. All experiments were repeated three times.

**Supplementary Figure 2.** LV-bFGF-NSCs increase neural stem cell survival and proliferation *in vitro*. **A)** Representative FACS analysis showing PI/Annexin V-FITC staining apoptotic cells induced by TG for 12 hours *in vitro*. Values represent the apoptosis rate statistics. **B)** Percentage of apoptotic cells induced by TG. **C-E)** At different concentrations of serum in culture, LV-bFGF-NSCs show increased proliferation of neural stem cells by MTT assay. Especially at low concentrations or free serum conditions, the proliferation rate is more significant in LV-bFGF-NSCs. * represents P < 0.05 and ** represents P < 0.01 versus the 1d group, # represents P < 0.05 and ## represents P < 0.01 versus NSCs and LV-GFP-NSCs group. Data are the mean values ± SEM. **F-G)** MTT assay results of different experimental groups treated with TG for 12 hours *in vitro*. * represents P < 0.05 or ** represents P < 0.01, # represents P < 0.05. Data are the mean values ± SEM. All experiments were repeated three times.

**Supplementary Figure 3.** In spinal cord injury, LV-5HRE-bFGF-NSCs maximize the promotion of primary embryonic neural stem cell differentiation and cell survival. **A)** Immunofluorescence staining results of nestin, 60 days after NSCs transplantation in SCI. Transplanted NSCs express red fluorescent signals (CM-DiI). Green fluorescence represents nestin. The nuclear is labelled by hoechst (blue). The arrows indicate the typical morphology of nestin. A boxed region illustrates a representative region with high power images. Scale bar = 100 μm. **B)** Immunofluorescence staining of the cross-section of spinal cord injury center. **C)** Quantification of the percentage of differentiated cells by nestin staining verse CM-DiI signals. * represents P < 0.05 and ** represents P < 0.01 , # represents P < 0.05. Data are the mean values ± SEM, n = 3.

**Supplementary Figure 4.** In spinal cord injury, LV-5HRE-bFGF-NSCs maximize the promotion of primary embryonic neural stem cell survival and neuronal differentiation. **A)** Immunofluorescence staining results of NeuN, 60 days after NSCs transplantation in SCI. Transplanted NSCs express red fluorescent signals (CM-DiI). Green fluorescence represents NeuN. The nuclear is labelled by hoechst (blue). The arrows indicate the typical morphology of NeuN. A boxed region illustrates a representative region with high power images. Scale bar = 100 μm. **B)** Immunofluorescence staining of the cross-section of spinal cord injury center. **C)** Quantification of the percentage of NeuN positive cells verse CM-DiI signals. * represents P < 0.05 and ** represents P < 0.01, # represents P < 0.05. Data are the mean values ± SEM, n = 3.

**Supplementary Figure 5.** LV-bFGF-NSCs promote neural stem cells from the spinal cord injury center to both sides. **A)** LV-bFGF-NSCs transplantation into the spinal cord injury center. Horizontal section immunolabelled with CM-DiI signals indicates that implanted cells survive well and distribute through the lesion cavity, scale bar = 500 μm. Rostral is to left, and caudal is to right. White cross lines represent the five cross-sections of spinal cord injury. (a1-e1) Five white lines corresponding to the cross sectional view of the spinal cord, scale bar = 200 μm. (a2-e2) A boxed region illustrates a representative region with high power images, scale bar = 100 μm. **B)** LV-GFP-NSCs transplantation into the spinal cord injury center. Horizontal section immunolabelled with CM-DiI signals indicates that implanted cells survive well and distribute through the lesion cavity, scale bar = 500 μm. Rostral is to left, and caudal is to right. White cross lines represent the five cross-sections of spinal cord injury. (f1-j1) Five white lines corresponding to the cross sectional view of the spinal cord, scale bar = 200 μm. (f2-j2) A boxed region illustrates a representative region with high power images, scale bar = 100 μm. **C)** Quantification of CM-DiI labelled positive after transplantation of neural stem cells per mm^2^. ** represents P < 0.01, ## represents P < 0.01. Data are the mean values ± SEM, n = 3.

**Supplementary Figure 6.** Hypoxia regulated LV-5HRE-bFGF-NSCs minimize the inhibition of autophagy. **A）**The protein expressions of Beclin-1, P62 and LC3 treated with sham group, SCI group, LV-GFP-NSCs group, LV-bFGF-NSCs group, LV-5HRE-GFP-NSCs group and LV-5HRE-bFGF-NSCs group in 14 days after SCI. GAPDH was used as the loading control and for band density normalization. **B-D)** The optical density analysis of Beclin-1, P62 and LC3 protein. * represents P < 0.05 and ** represents P < 0.01 versus SCI group, # represents P < 0.05 and ## represents P < 0.01, data are the mean values ± SEM, n = 3. **E)** The protein expression of Beclin-1, P62, LC3II/LC3I in the different neural stem cell groups treated rapamycin (RAPA), and with RAPA and 3-methyladenine (3-MA) under normal or hypoxia (pO_2_< 1%) condition for 12 hours. **F-H)** The optical density analysis of Beclin-1, P62 and LC3 protein. * represents P < 0.05 and ** represents P < 0.01 versus the NSCs+RAPA group, LV-GFP-NSCs+RAPA group and LV-5HRE-GFP-NSCs+RAPA group, ## represents P < 0.01, data are the mean values ± SEM. The data represent the results of three separate experiments.

**Supplementary Figure 7.** Inhibition of autophagy promotes functional recovery of spinal cord injury. **A)** Immunofluorescence staining results of LC3 after 3-MA or rapamycin (RAPA) plus 3-MA treatment for 14 days in SCI. Green fluorescence represents LC3. The nuclear is labelled by hoechst (blue). Scale bar = 200 μm. **B)** The protein expressions of Beclin-1, P62 and LC3 in 14 days after SCI. GAPDH was used as the loading control and for band density normalization. **C-E)** The optical density analysis of Beclin-1, P62 and LC3 protein. ** represents P < 0.01, data are the mean values ± SEM, n=3. **F)** Immunofluorescence staining results of NSCs treated with RAPA or RAPA plus 3-MA *in vitro*. Red fluorescence represents LC3. The nuclear is labelled by hoechst (blue). Scale bar = 20 μm. **G)** The protein expressions of Beclin-1, P62 and LC3 in 12 hours after SCI. GAPDH was used as the loading control and for band density normalization. **H-J)** The optical density analysis of Beclin-1, P62 and LC3 protein. ** represents P < 0.01, data are the mean values ± SEM, All experiments were repeated three times. **K)** The BBB scores of sham group, SCI group, SCI+3-MA group and SCI+3-MA+RAPA group. The score of sham group was 21 points, which means normal locomotion. * represents P < 0.05 versus the SCI group. Data are the mean values ± SEM, n= 6. **L)** The inclined plane test scores of the different groups. * represents P < 0.05 versus the SCI group. Data are the mean values ± SEM, n= 6.

**Supplementary Figure 8.** RAPA-induced apaoptosis was significantly reduced by 3-MA and by the expression of bFGF in primary neural stem cells. **A)** FACS result by PI/annexin V-FITC staining for cell apoptosis analysis treated with RAPA, and RAPA compound with 3-MA, and 3-MA for 12 hours under normal or hypoxia (pO_2_< 1%) condition. Values represent the apoptosis rate. **B-G)** Statistical result of apoptosis rates. ** represents P < 0.01. Data are the mean values ± SEM. All experiments were repeated three times.

Supplemental Figure 1


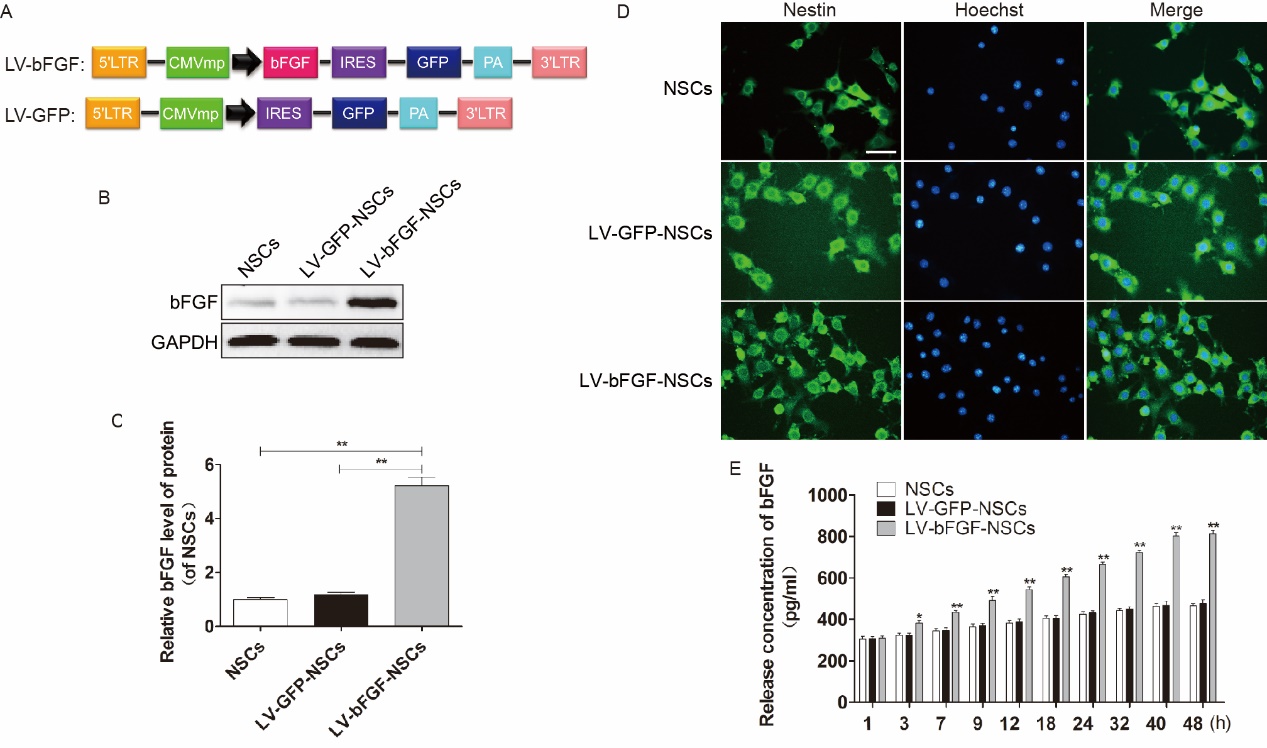


Supplemental Figure 2


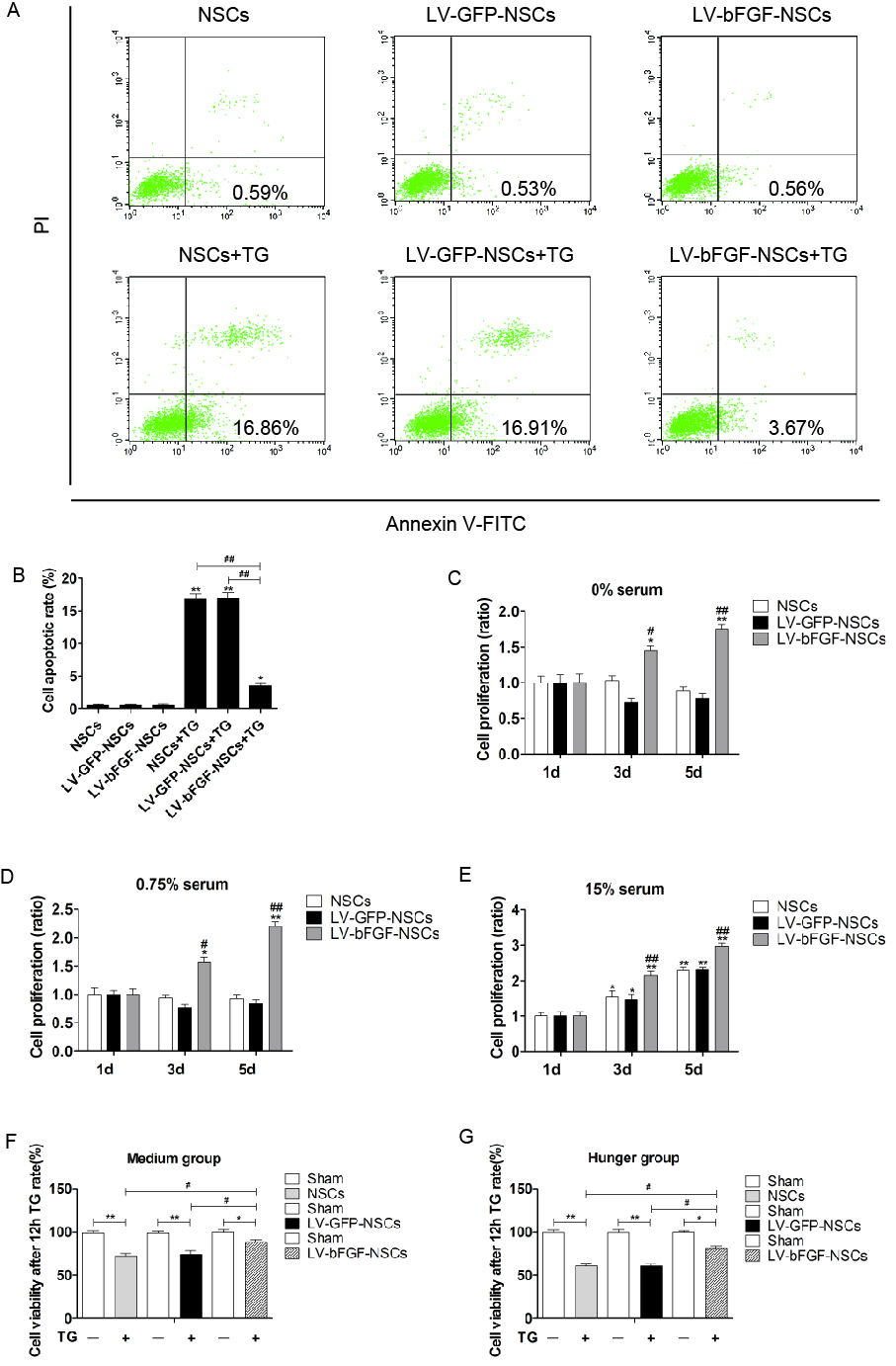


Supplemental Figure 3


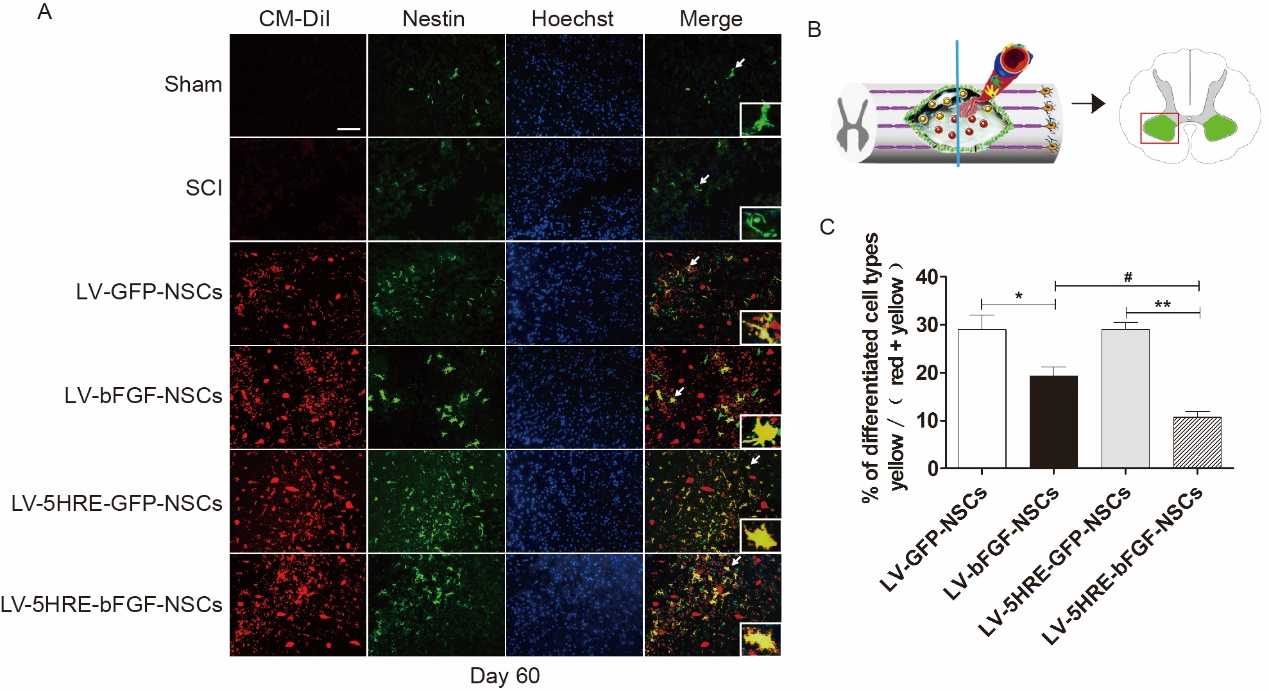


Supplemental Figure 4


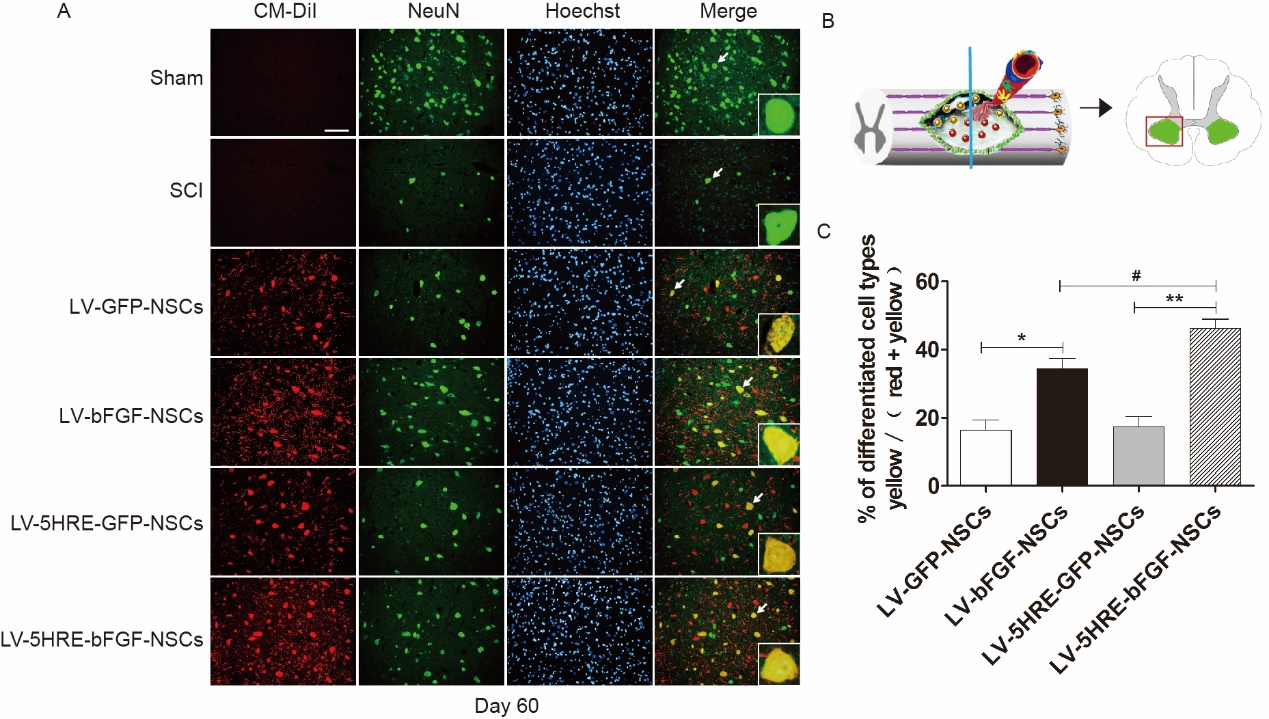


Supplemental Figure 5


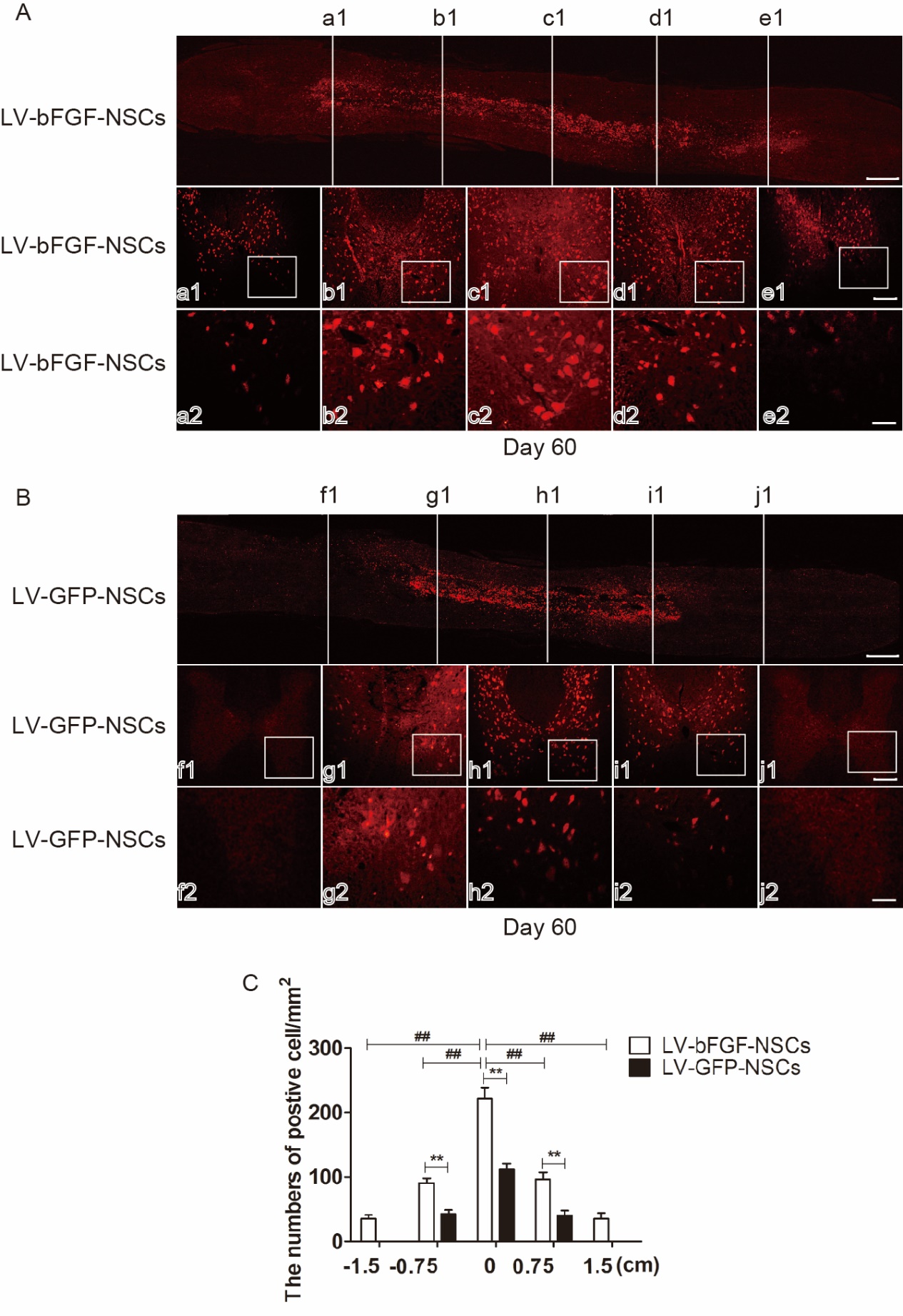


Supplemental Figure 6


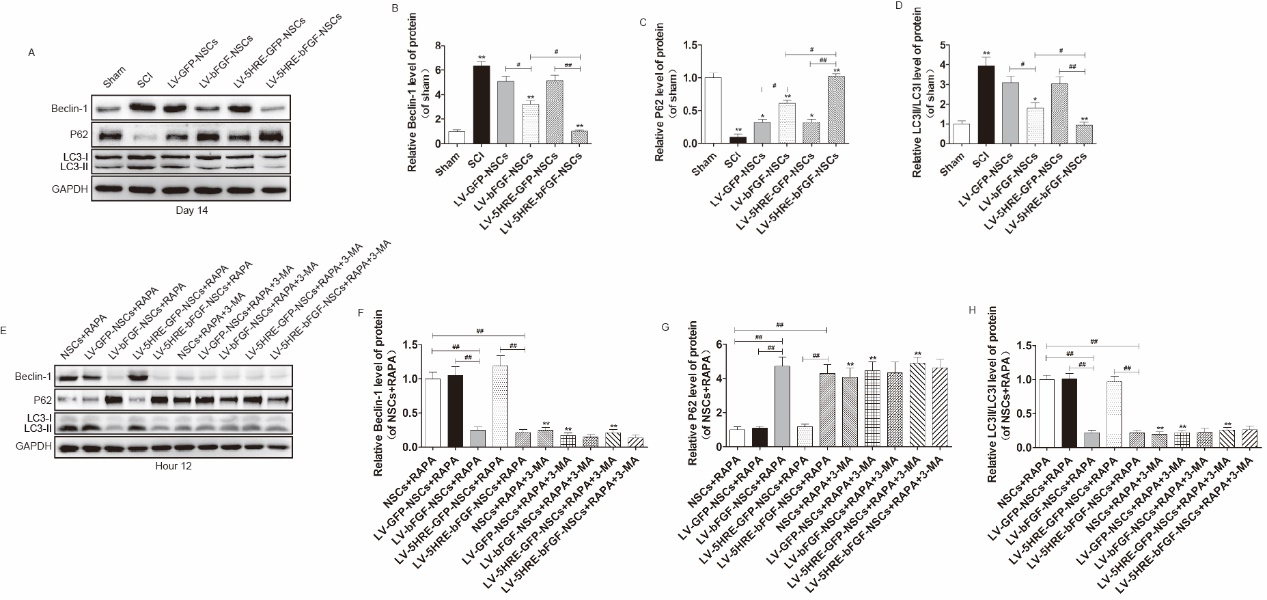


Supplemental Figure 7


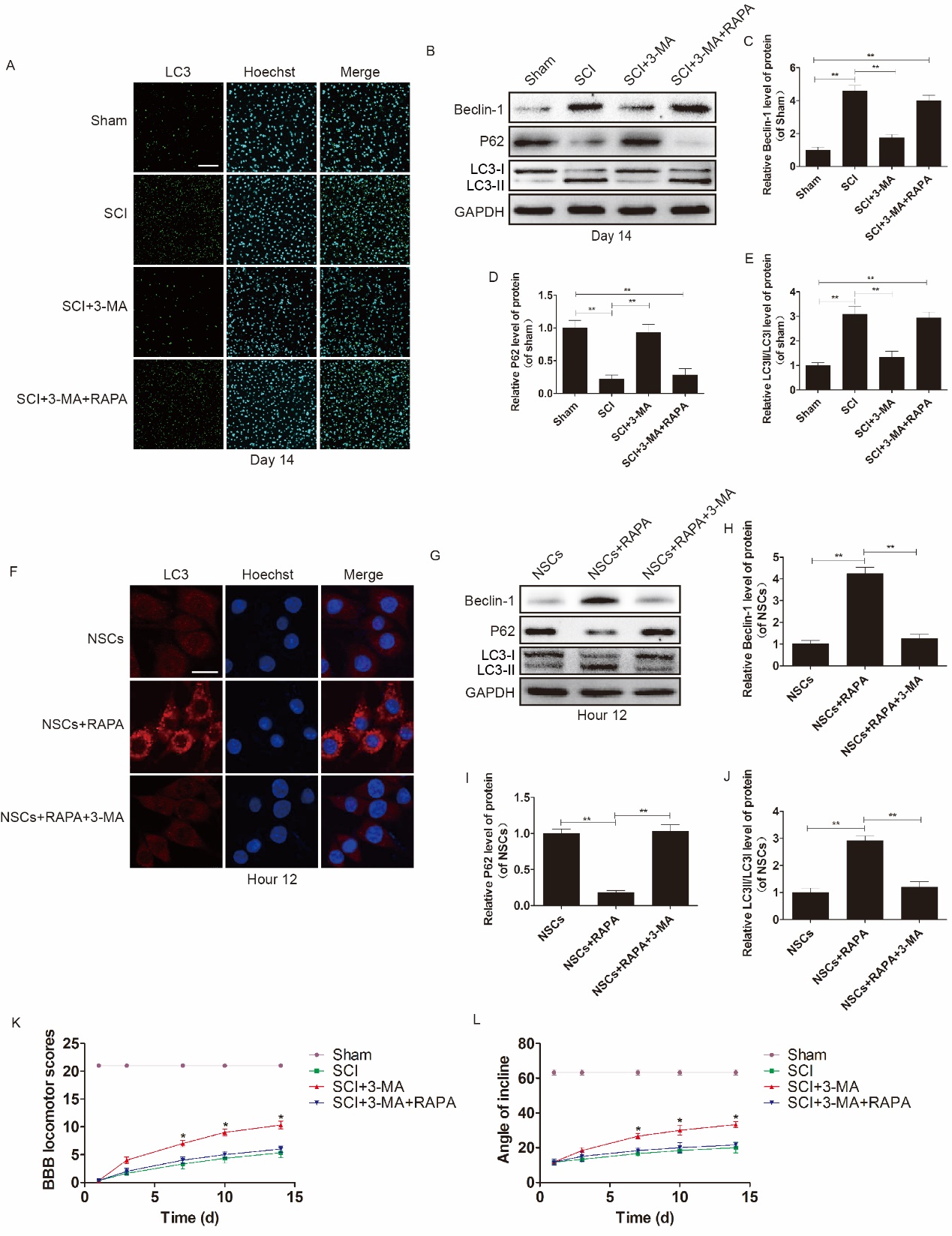


Supplemental Figure 8


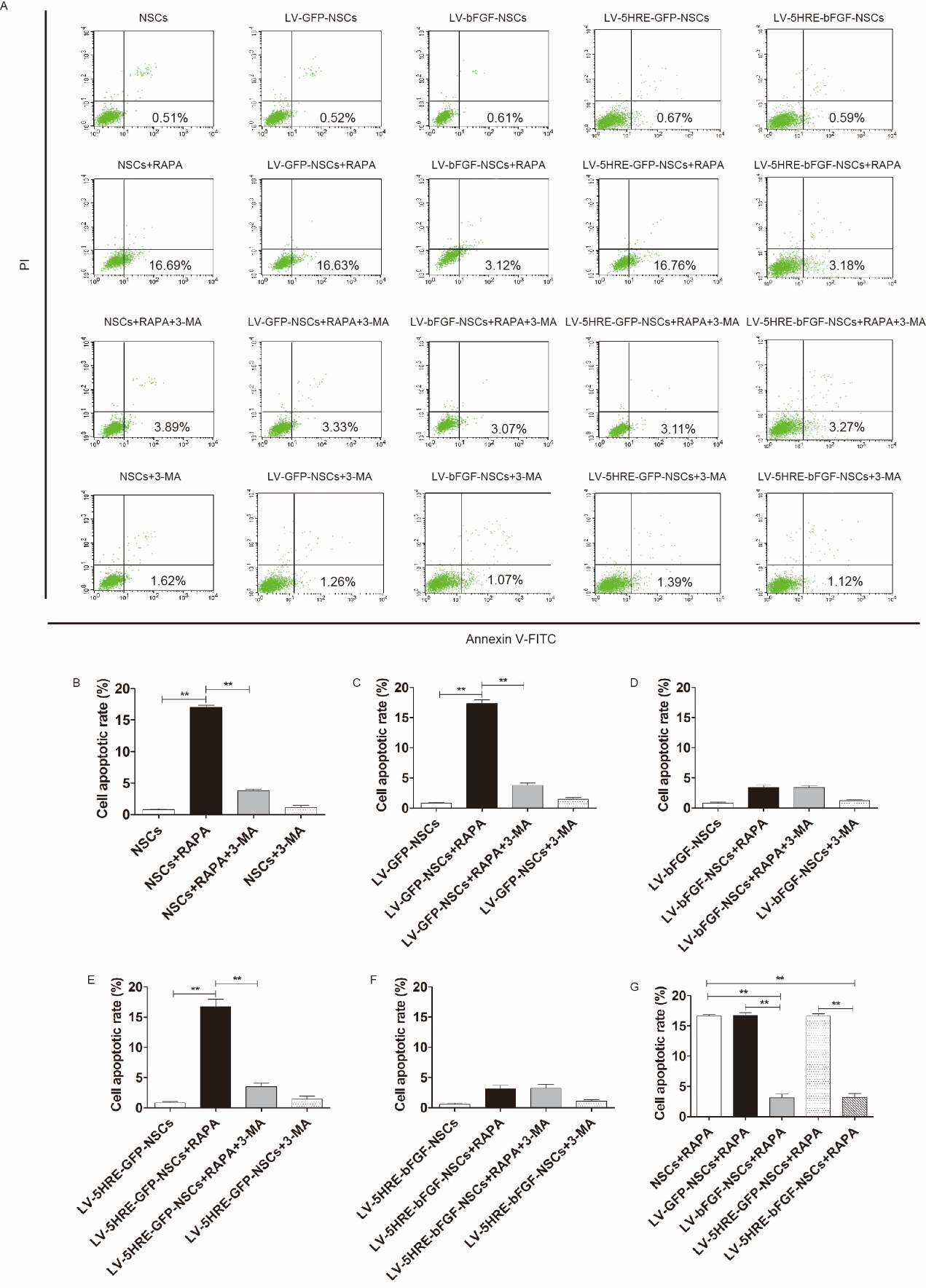

Supplement: Supplementary file 1 — Figure S1 Preparation and characterization of the LV‐GFP‐NSCs and LV‐bFGF‐NSCs. A) Schematic drawing of the lenti‐bFGF and lenti‐hrGFP vector constructs. B) Primary NSCs, LV‐GFP‐NSCs and LV‐bFGF‐NSCs were successfully generated showing bFGF expression by western blotting. C) The optical density analysis of bFGF protein. ** represents P < 0.01 vs the NSCs group and LV‐GFP‐NSCs group, data are the mean values ± SEM. All experiments were repeated three times. D) Nestin staining for the identification of primary neural stem cells successfully generated. Scale bar = 100 μm. E) Levels of bFGF analyzed by ELISA at 0‐48 hours with cultures of NSCs, LV‐GFP‐NSCs, LV‐bFGF‐NSCs group in vitro. * represents P < 0.05 and ** represents P < 0.01 vs the NSCs and LV‐GFP‐NSCs group, data are the mean values ± SEM. All experiments were repeated three times. Figure S2. LV‐bFGF‐NSCs increase neural stem cell survival and proliferation in vitro. A) Representative FACS analysis showing PI/Annexin V‐FITC staining apoptotic cells induced by TG for 12 hours in vitro. Values represent the apoptosis rate statistics. B) Percentage of apoptotic cells induced by TG. C‐E) At different concentrations of serum in culture, LV‐bFGF‐NSCs show increased proliferation of neural stem cells by MTT assay. Especially at low concentrations or free serum conditions, the proliferation rate is more significant in LV‐bFGF‐NSCs. * represents P < 0.05 and ** represents P < 0.01 vs the 1d group, # represents P < 0.05 and ## represents P < 0.01 vs NSCs and LV‐GFP‐NSCs group. Data are the mean values ± SEM. F‐G) MTT assay results of different experimental groups treated with TG for 12 hours in vitro. * represents P < 0.05 or ** represents P < 0.01, # represents P < 0.05. Data are the mean values ± SEM. All experiments were repeated three times. Figure S3. In spinal cord injury, LV‐5HRE‐bFGF‐NSCs maximize the promotion of primary embryonic neural stem cell differentiation and cell survival. A) Immunofluorescence stainin [file SCT3-9-603-s001.docx]
